# Exploring the Regulatory Function of NGAL in MMP-9 Complexes

**DOI:** 10.34133/csbj.0006

**Published:** 2026-03-30

**Authors:** Łukasz Charzewski, Krystiana A. Krzyśko

**Affiliations:** Department of Biophysics, Faculty of Physics, University of Warsaw, Pasteura 5, 02-093 Warsaw, Poland.

## Abstract

Matrix metalloproteinase 9 (MMP-9) is an enzyme involved in the degradation of the extracellular matrix and in numerous physiological and pathological processes. One of the less understood regulatory mechanisms of MMP-9 is its interaction with neutrophil gelatinase-associated lipocalin (NGAL), leading to the formation of a covalent or noncovalent complex, which may affect the enzyme’s activity, its stability, and binding by tissue inhibitors of metalloproteinases (TIMPs), in particular TIMP-1. In this study, molecular modeling was used to investigate the structural basis of NGAL’s regulatory effect. Covalent and noncovalent MMP-9/NGAL complexes were constructed and analyzed, as well as the tetrameric inhibitory complex MMP-9/NGAL/TIMP-1/MMP-9. Results indicate that NGAL stabilizes the structure of the hemopexin domain of MMP-9, protecting it from autodegradation. Furthermore, the identified protein interactions may explain the experimentally observed increased affinity of TIMP-1 for the MMP-9/NGAL complex. Comparison with the terminal MMP-9/TIMP-1 complex shows that the presence of NGAL induces slight but reproducible changes in the inhibitory interfaces.

## Highlights

- NGAL stabilizes the MMP-9 hemopexin domain and protects it from autodegradation.
- NGAL enhances TIMP-1 binding to latent MMP-9.
- MMP-9/NGAL/TIMP-1/MMP-9 tetramer shows stable intermolecular interfaces.
- NGAL modulates the stability and spatial organization of latent MMP-9/TIMP-1 assemblies.

## Graphical Abstract

**Figure:**
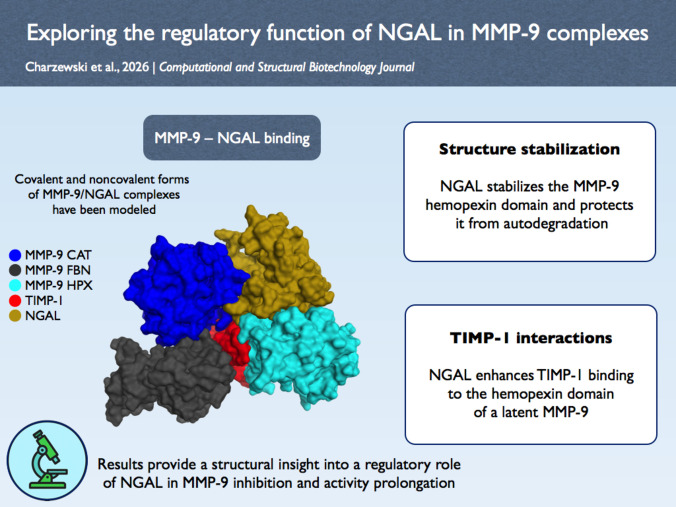

## Introduction

Matrix metalloproteinases (MMPs) are a family of zinc-dependent endopeptidases that play a key role in degradation and remodeling of the extracellular matrix (ECM) [1]. The ECM, a complex network of polysaccharides and proteins, provides tissues a structural support and even regulates cell functions [2]. Through the degradation of ECM components such as elastin, proteoglycans, and collagen, MMPs facilitate cellular migration, tissue remodeling, and angiogenesis [3]. Beyond the ECM remodeling, MMPs participate in a number of physiological processes [4]. They have the ability to activate growth factors and cytokines, influence cell signaling pathways, and modulate immune responses [5].

MMP enzymatic activity is tightly regulated at several levels, such as gene expression, activation of latent proenzymes, and deactivation by endogenous proteins such as tissue inhibitors of metalloproteinases (TIMPs) [1]. Understanding these mechanisms is crucial for developing therapeutic strategies to target their dysregulation in various diseases [6].

MMP-9 is an important member of the MMP family, participating in various physiological processes. Due to its ability to degrade a wide range of ECM components, MMP-9 takes part in normal tissue remodeling and repair processes, such as wound healing and bone development [7]. In these contexts, MMP-9’s actions facilitate cell migration and the creation of new blood vessels, crucial steps in the restoration of tissue integrity.

However, the MMP-9 activity can also contribute to pathological states. For example, in cancer progression, MMP-9’s ability to degrade the ECM enables the tumor invasion into surrounding tissues and their metastasis to distant organs [8]. MMP-9 is also involved in inflammatory conditions via the modulation of cytokines and chemokines, which are principal signaling molecules in the immune system [5]. MMP-9 is also involved in neurodegenerative disorders and cardiovascular diseases [4]. In the central nervous system, MMP-9’s involvement in synaptic plasticity indicates its role in learning and memory [9].

The most common MMP-9 form is a monomer. The molecular fragment of catalytic and fibronectin-like domains is connected to the hemopexin domain (HPX) by a long, flexible linker region, which is often referred to as an OG domain due to its strong O-glycosylation. The catalytic domain houses the active site responsible for cleaving ECM components, the fibronectin-like domain contributes to substrate recognition and binding, while the hemopexin is responsible for interactions with other proteins. The OG domain provides considerable conformational flexibility, enabling MMP-9 to interact with diverse regulatory molecules [10].

In contrast to the monomeric form, trimeric proMMP-9 has a distinctive circular arrangement. This unique configuration is stabilized by disulfide bonds as well as by noncovalent interactions between HPXs and the catalytic/fibronectin-like domains of adjacent subunits. In addition, the OG domain passes through the center of the circular structure, contributing to its stability. The trimeric form of proMMP-9 is believed to play a role in regulating the activity and bioavailability of MMP-9 [11].

There is a third form of MMP-9, in which the protein can be secreted as a heterodimer. Monocytes and neutrophils secrete it prebound in a complex with a neutrophil gelatinase-associated lipocalin (NGAL). Free NGAL, either monomeric or homodimeric, modulates the immune system by binding neutrophile-targeting chemoattractants. It can also be secreted by epithelial cells in the inflammation or neoplasia. Moreover, it is suggested that the MMP-9/NGAL complex may be a marker of a disease stage in some cancers. Its primary function remains unclear, though [12,13].

In the MMP-9/NGAL dimer, subunits are covalently linked through a disulfide bond involving Cys87 of NGAL and a cysteine of the HPX of MMP-9 [14]. This disulfide-linked MMP-9/NGAL heterodimer represents a physiological form of the complex observed in vivo and constitutes the structural model investigated in the present study. The specific cysteine of HPX has not been determined experimentally, but considering the involvement of Cys516 and Cys704 in the disulfide bridge in HPX, the strongest candidate seems to be the Cys674, which remains free in a MMP-9 monomer. It should be mentioned that the MMP-9/NGAL complex can form spontaneously, without the involvement of a disulfide bridge [15]. The MMP-9/NGAL complex is associated with an increase of MMP-9 enzymatic activity. Commonly, 2 mechanisms are discussed—promotion of proMMP-9 autoactivation by NGAL and protection from degradation [15,16].

Autoactivation of proMMP-9 is a proteolytic activation carried out by other MMP-9 molecules. In vitro studies indicate that the autoactivation is increased in the presence of the NGAL protein. For this to happen, the NGAL binding pocket should be unoccupied; adding peptides that occupy the NGAL cavity was found to interfere with this effect. It has been proposed that interactions between the MMP-9 propeptide and the NGAL cavity is the cause of the MMP-9 catalytic center exposure. This, in effect, enables MMP-9 to proteolytically cleave off the propeptide. This is done at the site Ala93*–*Met94, which is located at the C-terminal end of the propeptide’s third α-helix. An enzyme activated this way is 13 amino acids longer than the form activated by MMP-3. The enzymatic activity obtained by coincubation was linearly dependent on the NGAL concentration. However, at a proMMP-9:NGAL molar ratio of approximately 1:2.5, the enzymatic activity was just 10% greater than that of the control group, which lacked NGAL. These findings indicate that such a mechanism of activation might be physiologically irrelevant [17].

The proMMP-9/TIMP-1/NGAL complex can be isolated from heart muscle homogenates [18]. Its existence suggests that the TIMP-1 and NGAL binding sites on the HPX are nonoverlapping. In addition, TIMP-1 was found associated only with the proMMP-9/NGAL complex but not the proMMP-9 monomer. This may indicate a different pathway for the proMMP-9 monomer and the proMMP-9/NGAL dimer before secretion from the cell but also an increased association constant of TIMP-1 due to the NGAL presence on the HPX. It was also experimentally shown that the proMMP-9/TIMP-1/NGAL complex can inhibit active MMP-9. In this system, TIMP-1 remains associated with the trimer, leading to the experimental observation of the proMMP-9/TIMP-1/NGAL/MMP-9 tetramer [19]. This arrangement suggests that NGAL is positioned on the HPX of proMMP-9 in a manner that preserves the inhibitory function of TIMP-1. Interestingly, bound NGAL also shields MMP-9 from autodegradation, thereby enabling it to retain enzymatic activity for an extended period [15]. Under pathological conditions, the proMMP-9/NGAL complex can be expressed by hematopoietic cells of the bone marrow, which makes it a valuable prognostic indicator for patients’ condition in various types of leukemia [14].

However, the structural determinants of how NGAL affects MMP-9 interdomain interactions and its binding to TIMP-1 remain poorly understood. In this study, we aimed to characterize the structural basis of NGAL-mediated modulation of MMP-9 activity and its interactions with TIMP-1, using molecular modeling and simulation methods. In particular, this study focuses on the NGAL-dependent modulation of protein–protein interfaces within MMP-9-containing complexes, rather than on mechanisms of MMP-9 activation or catalysis.

Importantly, current evidence supports 2 well-defined regulatory interactions involving MMP-9, namely, the formation of the MMP-9/NGAL complex and the inhibition of MMP-9 by TIMP-1. In this study, we define the regulatory role of NGAL as its ability to modulate the stability and spatial organization of MMP-9/TIMP-1 assemblies, rather than as a direct inhibitor of MMP-9 catalytic activity. In contrast, a direct molecular interaction between NGAL and TIMP-1 has not been demonstrated to date, and the integration of these 2 regulatory pathways into a single molecular framework remains unresolved. Therefore, the structural characterization of higher-order assemblies involving MMP-9, NGAL, and TIMP-1 represents an important missing link in our understanding of MMP-9 regulation in inflammatory and tissue injury-associated conditions.

Here, we address this gap by means of molecular modeling and molecular dynamics (MD) simulations, providing a comparative atomistic characterization of the MMP-9/NGAL, MMP-9/TIMP-1, and MMP-9/NGAL/TIMP-1/MMP-9 assemblies and assessing whether NGAL induces specific conformational and interfacial rearrangements relevant for MMP-9 regulation.

## Materials and Methods

For the NGAL protein, the crystal structure 1DFV (chain A) [13] was selected, as it represents the native, nonengineered NGAL structure and does not contain any ligands bound in the binding pocket, which could potentially bias protein–protein docking results. The structure was prepared for docking by removing the covalently linked saccharide moiety.

Considering that full-length MMP-9 is a large and highly flexible multidomain protein and that the HPX is directly involved in NGAL binding, the interface search was restricted to the HPX of MMP-9. For this domain, its only available crystallographic structure, 1ITV (chain A) [20], was selected, which allowed us to focus on an experimentally resolved NGAL-binding region and to avoid uncertainties associated with full-length structure modeling.

The MMP-9 hemopexin structure required additional preparation, including minor adjustments of the Gln675–Arg677 region and rotation of the His662 side chain in order to expose the Cys674 residue, which was subsequently oriented toward the solvent to enable formation of the experimentally suggested disulfide bond with NGAL. Both structures were finally optimized by energy minimization prior to docking.

The docking was carried out using protein–protein docking protocol available in MOE2020 [21]. A total of 10,000 initial positions were generated and reduced to 1,000 best-scored models. After the refinement with energy minimization, 100 models were retained based on a generalized Born / volume integral scoring function. The best-scored model was modified by rotating Cys674 of MMP-9 and Cys87 of NGAL side chains toward each other, which reduced their distance to 6.7 Å. This distance was further reduced to 3.9 Å by introducing harmonic restraint of 1 kcal/mol/Å force constant with equilibrium in a distance of 3 Å between the S atoms followed by the energy minimization. Then, the restraints were removed, the disulfide bridge was introduced to the system, and another minimization was run, resulting in 2.05-Å S–S distance. The disulfide bond was introduced a posteriori and treated as a predefined covalent bond in the molecular mechanics model. The noncovalent complex was obtained after completion of the covalent complex MD simulations by removal of the disulfide bond and subsequent energy minimization of the structure retained from the last frame of #1 MD simulation (MD procedure described below). For molecular manipulation involving structure preparation, docking, and energy minimization, the AMBER10:EHT force field [22,23] and MOE2020 software were used. The target of 0.01 kcal/mol/Å^2^ energy gradient was set for minimization simulations.

MMP-9/NGAL/TIMP-1/MMP-9 tetramer was modeled using our previously published model of a terminal-inhibitory MMP-9/TIMP-1 complex [24], which was originally derived from the experimentally resolved MMP-2–TIMP-2 complex structure (Protein Data Bank ID: 1GXD). The OG domain was removed from the model, and the NGAL structure was introduced using the structural alignment of covalent MMP-9/NGAL HPX complex. To remove steric clashes, the unstructured NGAL amino acids Gln1–Ala10 were slightly rotated away from the MMP-9 catalytic domain and minimized.

In order to relax the structures and identify the most stable interactions, all modeled systems were placed in an explicit solvent using the TIP3 water model with 0.05 M NaCl concentration. Lower than physiological ionic strength was used to reduce electrostatic screening and facilitate identification of key interfacial interactions within the limited simulation time scale. All simulations were run in a cuboid box with periodic boundary conditions**.** The system energy was minimized, followed by 10-ns MD simulations in NAMD 2.13 [25] with CHARMM27 force field [26]. MD simulations were run in isothermal–isobaric ensemble with 1-fs timestep, at a pressure of 1.01325 bar controlled by Nosé–Hoover Langevin piston algorithm, and at a temperature of 310 K controlled by Langevin dynamics.

For the covalent and noncovalent MMP-9/NGAL dimeric complexes, 5 independent MD replicas were performed for each system, whereas 3 independent replicas were carried out for the MMP-9/NGAL/TIMP-1/MMP-9 tetrameric complex. In all cases, the replicas were started from the same minimized structure and simulated using identical parameters, differing only in the initial atomic velocities assigned according to a Maxwell–Boltzmann distribution.

A larger number of replicas were used for the dimeric systems in order to robustly characterize the NGAL–MMP-9 HPX interface, which constitutes the primary focus of this study. For the tetrameric MMP-9/NGAL/TIMP-1/MMP-9 assembly, 3 replicas were considered sufficient, as the TIMP-1–MMP-9 interaction interface has been shown to be highly reproducible in our previous work, and the NGAL–MMP-9 interface displayed similarly stable behavior in the present study.

Root-mean-square deviation (RMSD) was calculated in Visual Molecular Dynamics (VMD) 1.9.3 [27] using backbone atoms selection, and subunit contact areas were calculated using SASA in VMD. Pairwise RMSD matrices were calculated in VMD 1.9.3 using Cα atoms after least-squares superposition, by computing RMSD values between all pairs of trajectory frames. RMSF values were calculated in VMD 1.9.3 for Cα atoms as time-averaged positional fluctuations of each residue around its mean position over the analyzed part of the trajectory. RMSD and RMSF analyses were performed using only the equilibrated part of each trajectory. RMSD profiles were calculated for the entire trajectories starting from the initial simulation frame. In contrast, RMSF profiles were calculated only for the stabilized parts of the trajectories, and the corresponding results are provided in the Supplementary Materials. Hydrogen bond frequencies were analyzed using hbonds 1.2 VMD plugin with a donor–acceptor distance limit of 3.5 Å and donor–hydrogen–acceptor angle of 120° to 180°. Contact frequencies were analyzed using fResPair procedure, available at the VMD website, with side chain selection and distance threshold of 5 Å. Contacts were analyzed starting from the reported time of system relaxation to the end of the simulation. Contact occupancies were averaged across simulation replicas, from which we selected amino acid pairs forming electrostatic and van der Waals interactions. We interpret interactions ≥70% as stable, ≥50% as frequent, ≥20% as intermittent, and ≥10% as transient. Throughout this study, interactions and structural changes described as substantial refer to effects that are consistently observed across independent simulations and supported by the applied interaction-frequency criteria as well as by interface energy and contact-area analyses. The interaction energy was determined with the single trajectory scheme of molecular mechanics-generalized Born surface area [28,29] method in NAMD 2.13. The molecular mechanics-generalized Born surface area interaction energies account only for noncovalent interaction terms and solvation contributions and do not include covalent bond energies.

Importantly, the aim of the performed MD simulations was to verify the structural stability of the docked models and to identify the most probable and persistent protein–protein contacts, rather than to investigate the global dynamics of the systems. The 10-ns simulations were used as a relaxation and short structural equilibration stage of the complexes. Interaction networks were derived exclusively from trajectory fragments after RMSD stabilization, which allowed elimination of artifacts originating directly from the docking procedure. In this context, the analyzed contacts represent local and short-time scale interactions within protein–protein interfaces rather than long-time scale conformational transitions.

An overview of the complete computational workflow, including structure acquisition, preparation, docking, complex construction, and MD simulations, is provided in the Supplementary Materials (Section S1).

## Results

### MMP-9 HPX/NGAL docking

The docking run generated 99 distinct models. The obtained evaluation function values ranged from −55.33 to −18.05 kcal/mol. The median was −34.43 kcal/mol. The best-scoring model had a score approximately 8 kcal/mol lower than the next one. Such a difference suggests that this model is particularly interesting, and its interface appears to be well structured.

In the models, the distances between the sulfur atoms of Cys674 in the HPX of MMP-9 and Cys87 of NGAL were measured. These values ranged from 9.6 to 38.5 Å, with a median of 20.1 Å and the 10th and 90th percentiles being 13.3 and 28.4 Å, respectively.

Only 2 results (ranked 1 and 28) showed the expected distance below 10 Å: model 1 with 9.7 Å and model 28 with 9.6 Å. Model 1 was notably more favorable in terms of energy evaluation (by 15.75 kcal/mol) than model 28, and for this reason, it was used as the basis to design the biologically most important model of the MMP-9 HPX complex with NGAL—a complex in the covalent form.

### Covalent MMP-9 HPX/NGAL complex

The model was modified by introducing disulfide bonds and then subjected to MD simulation. The RMSD profiles show rapid relaxation followed by stable fluctuations after approximately 3 ns of simulation (see Section S2). Based on the trajectories, the interaction energy (#1: −54.62 ± 0.20 kcal/mol; #2: −42.66 ± 0.16 kcal/mol; #3: −40.45 ± 0.15 kcal/mol; #4: −53.25 ± 0.18 kcal/mol; #5: −57.86 ± 0.18 kcal/mol) and contact surface area (#1: 1,014.62 ± 1.24 Å^2^; #2: 883.5 ± 1.24 Å^2^; #3: 923.51 ± 1.11 Å^2^; #4: 922.28 ± 1.20 Å^2^; #5: 972.51 ± 1.52 Å^2^) between the subunits of the complex in the individual simulations were determined. The average interaction energy is −48.47 ± 3.54 kcal/mol, and the average contact surface area is 940.25 ± 22.60 Å^2^.

**Fig. 1.**
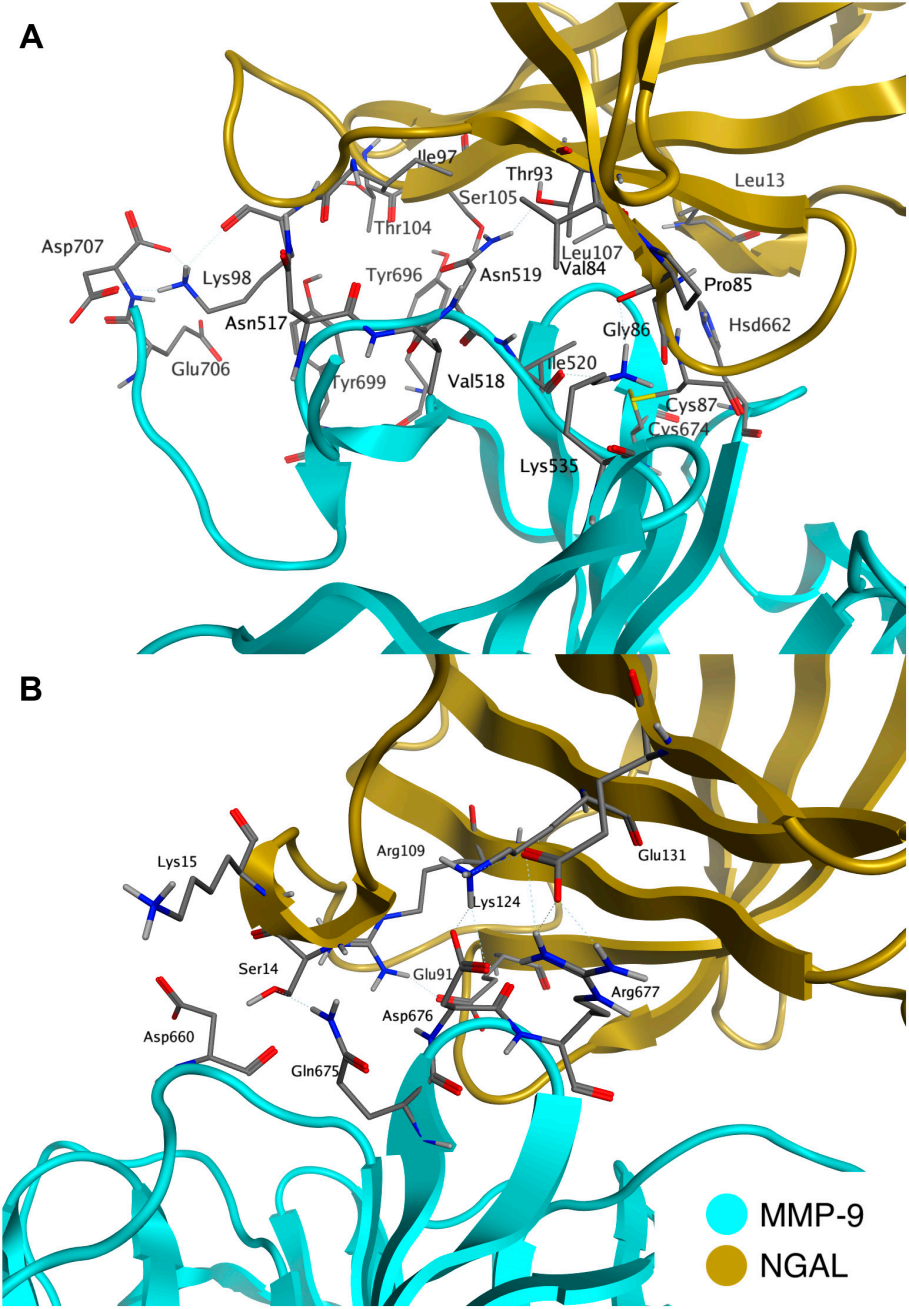
Interactions stabilizing the covalent MMP-9/NGAL complex: (A) interactions located in the vicinity of the Cys87–Cys674 bond and the N-terminal region of MMP-9 and (B) other interactions located further away from these sites. The structure shown represents a representative snapshot from the equilibrated MD simulation.

Trajectory analysis enabled the identification of 20 residue pairs exhibiting substantial interactions (hydrogen bonds, electrostatic interactions, and van der Waals interactions) that stabilize the complex (Table 1 and Fig. 1). Among them, 12 residue pairs formed stable interactions (occupancy ≥70%), 4 pairs formed frequent interactions (50% to 70%), and 4 pairs formed intermittent interactions (20% to 50%), according to the interaction frequency criteria defined in Methods. Residue pairs exhibiting interactions with occupancies below 20% were considered not relevant for complex stabilization and therefore are not reported, unless presented for comparison purposes with a noncovalent system.

**Table 1.** Interactions between the HPX of MMP-9 and the NGAL protein in covalent and noncovalent heterodimers. Interaction type notation: H, hydrogen bond; V, van der Waals interaction; E, electrostatic interaction; S, disulfide bridge. Interaction occupancy, measured since the reported relaxation time, is presented as a mean ± standard error of the mean (SEM) from all simulation replicas. Hydrogen bond occupancy is presented in square brackets, and participating amino acids are denoted with D (donors) and A (acceptors).

MMP-9	NGAL	Interaction type	Covalent system occupancy (%)	Noncovalent system occupancy (%)
Asn517:A Asn517:D	Ile97:D Ile97:A	H H	[70.33 ± 12.13] [29.33 ± 14.25]	[28.56 ± 18.5] [46.7 ± 10.62]
Val518	Val84	V	99.56 ± 0.37	69.8 ± 13.38
Val518	Thr93	V	89.92 ± 9.33	57.1 ± 18.31
Asn519:A Asn519:D	Thr93:D Thr93:A	H H	[9.07 ± 7.4] [15.21 ± 13.61]	[18.96 ± 18.89] [34.42 ± 15.3]
Asn519:D Asn519:A	Ser105:A Ser105:D	H H	[14.63 ± 14.02] [55.44 ± 15.9]	[18.78 ± 13.19] [25.08 ± 10.92]
Ile520	Gly86	V	100.0 ± 0.0	99.54 ± 0.46
Ile520	Thr93	V	100.0 ± 0.0	100.0 ± 0.0
Ile520	Leu107	V	91.74 ± 6.09	95.94 ± 3.32
Lys535:D	Pro85:A	H	[98.69 ± 0.93]	[99.2 ± 0.35]
Lys538 Lys538:D	Asp61 Asp61:A	E H	53.04 ± 12.2 [6.07 ± 5.47]	80.9 ± 12.82 [40.34 ± 11.6]
Asp660 Asp660:A	Lys15 Lys15:D	E H	22.52 ± 9.62 [-]	60.56 ± 14.08 [31.88 ± 17.92]
His662:D	Glu91:D	H	[77.71 ± 18.77]	[59.36 ± 19.87]
His662:A	Arg109:D	H	[20.44 ± 10.72]	[35.58 ± 9.71]
Cys674	Cys87	S	100.0 ± 0.0	–
Gln675:A	Ser14:D	H	[40.09 ± 14.88]	[5.33 ± 3.08]
Asp676:A	Leu13:D	H	[79.53 ± 19.88]	[93.04 ± 3.92]
Asp676 Asp676:A	Lys124 Lys124:D	E H	100.0 ± 0.0 [100.0 ± 0.0]	100.0 ± 0.0 [100.0 ± 0.0]
Arg677 Arg677:D	Glu131 Glu131:A	E H	100.0 ± 0.0 [97.14 ± 1.0]	100.0 ± 0.0 [92.36 ± 3.75]
Tyr696	Thr104	V	62.32 ± 11.89	63.84 ± 16.82
Tyr699	Thr104	V	62.2 ± 12.02	57.36 ± 16.27
Asp707 Asp707:A	Lys98 Lys98:D	E H	83.22 ± 6.48 [71.33 ± 12.95]	58.54 ± 18.49 [43.74 ± 16.11]

### Noncovalent MMP-9 HPX/NGAL complex

The noncovalent complex model was obtained by removing the disulfide bond from the relaxed, described above model. The system was then subjected to MD simulation. The RMSD profile shows rapid relaxation followed by stable fluctuations after approximately 5 ns of simulation (see Section S3).

Based on the trajectories, the interaction energy (#1: −47.53 ± 0.18 kcal/mol; #2: −51.04 ± 0.27 kcal/mol; #3: −47.65 ± 0.20 kcal/mol; #4: −49.66 ± 0.26 kcal/mol; #5: −52.85 ± 0.34 kcal/mol) and contact surface area (#1: 888.74 ± 1.5 Å^2^; #2: 936.58 ± 3.47 Å^2^; #3: 955.26 ± 1.71 Å^2^; #4: 983.38 ± 2.99 Å^2^; #5: 983.2 ± 2.62 Å^2^) between the subunits of the complex were determined. The average interaction energy is −48.91 ± 0.91 kcal/mol, and the average contact surface area is 934.02 ± 19.38 Å^2^.

**Fig. 2.**
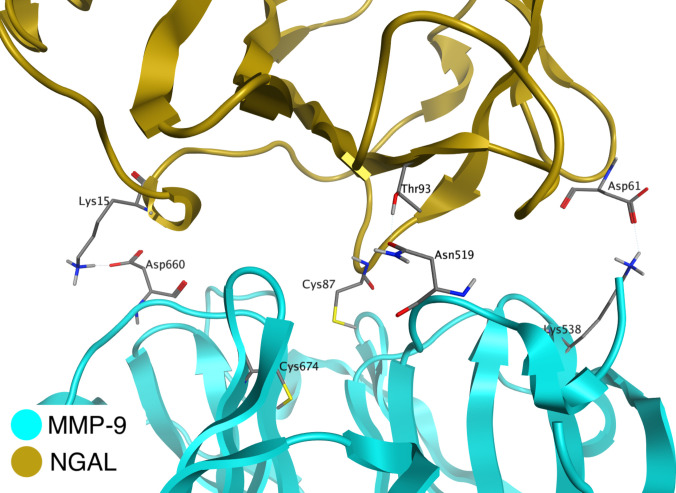
Additional interactions formed in the noncovalent MMP-9/NGAL dimer. The structure shown represents a representative snapshot from the equilibrated MD simulation.

The MD simulations revealed 19 interacting residue pairs (hydrogen bonds and electrostatic and van der Waals interactions) contributing to the stabilization of the noncovalent complex (Table 1 and Fig. 2). All of these residue pairs corresponded to interaction pairs identified in the covalent complex, although their interaction occupancies differed between the 2 systems. Among the 19 interacting residue pairs, 9 exhibited stable interactions (occupancy ≥70%). Seven of these stable residue pairs were the same as those classified as stable in the covalent complex.

### Inhibitory MMP-9/NGAL/TIMP-1/MMP-9 tetramer

The HPX complex with NGAL, stabilized covalently, was used for modeling a tetrameric system, as it is in this form that the proMMP9/NGAL complex is secreted by cells—neutrophils and monocytes [12,13]. Only after secretion of the proMMP-9/NGAL complex into the extracellular space TIMP-1 can bind to this complex, and subsequently, following proteolytic activation of proMMP-9, the catalytic domain of MMP-9 can be bound via TIMP-1 [11,15,19].

The RMSD profiles show that the MMP-9/NGAL/TIMP-1/MMP-9 systems reach stable fluctuations after approximately 3, 5, and 4.5 ns in simulations #1, #2, and #3, respectively (see Section S4). Based on the MD trajectories of the developed model, interaction energies between individual subunits of the complex were calculated. These results were compared with the values obtained for the corresponding interfaces in the terminal MMP-9/TIMP-1 complex (Table 2). The structural model of the terminal MMP-9/TIMP-1 complex was described previously [24]; however, energy calculations for individual molecular fragments allowing comparison with the present system were conducted for the first time in this study using a unified methodology. The trajectory analysis also allowed the identification of interactions contributing to the stabilization of the complex (Tables 3 and 4).

**Table 2.** The interaction energy values between the subunits of the MMP-9/NGAL/TIMP-1/MMP-9 complex, calculated from the point of system stabilization in MD simulations, presented as the mean ± SEM, in kcal/mol. Rows correspond to simulation replicas, and the averaged values are in the last row shown in bold.

	MMP-9 CAT/FBN–NGAL	MMP-9 HPX–NGAL	TIMP-1–NGAL	MMP-9 CAT/FBN–TIMP-1	MMP-9 HPX–TIMP-1	MMP-9/NGAL–TIMP-1
#1	−34.85 ± 0.11	−48.80 ± 0.16	−29.57 ± 0.21	−64.36 ± 0.20	−57.67 ± 0.16	−151.60 ± 0.37
#2	−20.22 ± 0.13	−58.87 ± 0.18	−35.17 ± 0.22	−62.22 ± 0.20	−59.71 ± 0.17	−157.12 ± 0.29
#3	−23.23 ± 0.13	−42.03 ± 0.18	−22.62 ± 0.19	−67.09 ± 0.24	−79.40 ± 0.21	−169.09 ± 0.29
**μ**	**−26.75 ± 4.58**	**−49.60 ± 4.65**	**−28.59 ± 3.65**	**−64.28 ± 1.36**	**−63.50 ± 6.26**	**−160.43 ± 5.08**

CAT, catalytic domain; fibronectin domain, FBN.

**Table 3.** Interactions identified between TIMP-1 and NGAL proteins in MMP-9/NGAL/TIMP-1/MMP-9 tetramer. Interaction type designations: H, hydrogen bond; V, van der Waals interaction; E, electrostatic interaction. Interaction occupancy, measured since the reported relaxation time, is presented as a mean ± SEM from all simulation replicas. Hydrogen bond occupancy is presented in square brackets, and participating amino acids are denoted with D (donors) and A (acceptors).

TIMP-1	NGAL	Interaction type	Contact occupancy (%)
Pro6	Leu7	V	28.93 ± 24.98
Arg75 Arg75:D	Asp2 Asp2:A	E H	47.73 ± 20.35 [23.8 ± 21.63]
Arg75 Arg75:D	Asp6 Asp6:A	E H	71.43 ± 24.53 [40.25 ± 26.48]
Thr98	Leu7	V	99.27 ± 0.64
Gln153	Glu131	H	[66.17 ± 32.81]
Ser155:D Ser155:A	Ala10:A Ala10:D	H H	[55.3 ± 28.36] [30.15 ± 11.05]
Lys157	Pro9	V	100.0 ± 0.0
Gly158	Pro12	V	49.73 ± 25.18
Phe159	Pro12	V	100.0 ± 0.0
His163:A	Lys15:D	H	[56.46 ± 27.67]

**Table 4.** Interactions identified between MMP-9 and NGAL proteins within the MMP-9/NGAL/TIMP-1/MMP-9 complex. Interaction type notation: H, hydrogen bond, V, van der Waals interaction, E, electrostatic interaction, S, disulfide bridge. Interaction occupancy, measured since the reported relaxation time, is presented as a mean ± SEM from all simulation replicas. Hydrogen bond occupancy is presented in square brackets, and participating amino acids are denoted with D (donors) and A (acceptors).

	MMP-9	NGAL	Interaction type	Contact occupancy (%)
MMP-9 catalytic domain	Asp410	Gln1	E	29.97 ± 4.76
	Asp410:A	Ser3:D	H	[11.7 ± 5.86]
	His411:D	Ser5:A	H	[17.1 ± 17.1]
	Val414	Ala40	V	76.63 ± 16.05
	Pro415	Ile8	V	99.7 ± 0.3
	Pro415:A	Arg130:D	H	[99.56 ± 0.22]
	Pro415	Tyr132	V	71.73 ± 14.13
	Pro415	Phe133	V	76.4 ± 21.19
	Glu416 Glu416:A	Lys125 Lys125:D	E H	95.73 ± 3.14 [43.65 ± 27.83]
	Ala417:A	Arg130:D	H	[62.69 ± 13.93]
	Tyr420	Leu7	V	100.0 ± 0.0
	Tyr420	Ile8	V	98.67 ± 1.33
	Pro421	Leu7	V	100.0 ± 0.0
	Met422	Leu7	V	100.0 ± 0.0
	Phe425:A	Arg130:D	H	[95.2 ± 2.85]
	Glu427:A	Ser127:D	H	[51.88 ± 16.47]
MMP-9 hemopexin domain	Asn517:D	Ile97:A	H	[39.57 ± 24.96]
	Asn519:A	Thr93:D	H	[28.73 ± 15.92]
	Asn519:D	Ser105:A	H	[10.09 ± 8.93]
	Ile520	Gly86	V	99.87 ± 0.09
	Ile520	Thr93	V	100.0 ± 0.0
	Ile520	Leu107	V	98.17 ± 1.83
	Lys535:D	Pro85:A	H	[99.42 ± 0.39]
	Asp660 Asp660:A	Lys15 Lys15:D	E H	81.3 ± 18.7 [71.84 ± 27.74]
	His662:D	Glu91:A	H	[79.14 ± 16.05]
	Cys674	Cys87	S	100.0 ± 0.0
	Gln675:D Gln675:A	Ser14:A Ser14:D	H H	[45.17 ± 23.6] [41.61 ± 29.25]
	Asp676:A	Leu13:D	H	[99.98 ± 0.02]
	Asp676 Asp676:A	Lys124 Lys124:D	E H	100.0 ± 0.0 [100.0 ± 0.0]
	Arg677:D	Asn129:A	H	[48.69 ± 23.52]
	Arg677 Arg677:D	Glu131 Glu131:A	E H	100.0 ± 0.0 [100.0 ± 0.0]
	Tyr696:D	Asn129:A	H	[55.04 ± 9.37]
	Asp707 Asp707:A	Lys98 Lys98:D	E H	93.43 ± 4.2 [58.41 ± 22.48]

In the analyzed system, the average interaction energy of the catalytic and fibronectin domains of MMP-9 with TIMP-1 is approximately 13 kcal/mol less favorable than for the analogous interface in the full inhibitory complex (−77.87 ± 1.94 kcal/mol; Table 5). This difference results from structural changes induced by NGAL in 2 interface regions—the β-turn Gln129–Arg130 and the Ser3–Pro9 fragment—which affect the local arrangement of MMP-9 and TIMP-1 residues, leading to their separation. Although these regions do not form stable contacts, the observed energy difference may be due, among other things, to solvation effects.

**Table 5.** Interaction energy between the subunits of the complete MMP-9/TIMP-1 inhibitory complex. The table also presents the interaction energy between fragments of the complex corresponding to fragmentary complexes. Rows correspond to simulation replicas, and the averaged values are in the last row shown in bold. Values are expressed in kcal/mol along with their associated uncertainties.

	MMP-9–TIMP-1	MMP-9 (CAT/FBN)–TIMP-1	MMP-9 HPX–TIMP-1
#1	−150.15 ± 0.71	−79.73 ± 0.59	−70.09 ± 0.68
#2	−146.47 ± 0.30	−75.11 ± 0.19	−68.59 ± 0.18
#3	−156.79 ± 0.24	−80.67 ± 0.19	−75.31 ± 0.17
**μ**	**−150.62 ± 4.30**	**−77.87 ± 1.94**	**−72.03 ± 2.35**

The interaction energy of the HPX of MMP-9 with TIMP-1 is approximately 9 kcal/mol less favorable than in the full inhibitory complex (−72.03 ± 2.35 kcal/mol), which results from the loss of 4 interactions with MMP-9 residues Tyr969 and Tyr699. Both tyrosines are shifted toward NGAL, forming transient hydrogen bonds with its residues. The energy value for this interface is similar to that previously determined for the fragmentary complex (−63.95 ± 3.23 kcal/mol).

It is also worth noting the interaction energy between the HPX of MMP-9 and the subunit composed of the catalytic and fibronectin domains. The average interaction energy between these fragments is −0.63 ± 1.13 kcal/mol, indicating random, negligible interactions that are not relevant for the stability of the model.

The simulation trajectories were analyzed to identify interactions between the subunits of the MMP-9/NGAL/TIMP-1/MMP-9 complex. The unstructured 5-residue N-terminus of NGAL moves freely, forming nonspecific contacts with the catalytic domain of MMP-9 and occasional interactions with TIMP-1. In contrast, the structured regions of NGAL form stable interfaces with the remaining subunits of the complex.

The interactions between NGAL and TIMP-1 are concentrated mainly in the N-terminal region of NGAL (Leu7–Lys15), which is responsible for both hydrogen bonding and hydrophobic interactions. Hydrophobic interactions involving Leu7, Pro9, and Pro12 of NGAL appear to be key for this interface. Leu7 fits into a cavity between TIMP-1 subdomains, while the other 2 residues support complex stabilization through contacts with neighboring surface regions of the inhibitor. Stable hydrophobic interactions observed in this region may contribute to TIMP-1 positioning and orientation within the tetrameric MMP-9/NGAL/TIMP-1/MMP-9 assembly.

The interactions between the HPX of MMP-9 and NGAL in the constructed tetramer remained structurally stable during the simulations (Table 4), consistent with the behavior observed for the simpler covalent dimer. It should be noted that the present model focuses on the structural organization of the assembly and does not address the catalytic activity of MMP-9 or the role of essential cofactors required for enzymatic function. In contrast, the interactions between NGAL and the catalytic domain of MMP-9 show greater variability and can be divided into 2 main regions: polar and hydrophobic. In the polar region, hydrogen bonding involving Arg130 of NGAL dominates, stabilizing the local structure of a loop in the catalytic domain. In the hydrophobic region, key roles are played by Leu7 and Ile8 of NGAL and Pro415 of the MMP-9 catalytic domain—the latter forms as many as 4 interactions with NGAL, serving as a hydrophobic contact center. From the perspective of NGAL, a similar function is performed by Leu7 and Ile8, which together interact with 4 residues of the MMP-9 catalytic domain. These 2 contact points likely play an important role in the formation of the MMP-9/NGAL/TIMP-1/MMP-9 tetramer, specifically in the association of the MMP-9/NGAL/TIMP-1 complex (containing the HPX) with the activated catalytic domain of MMP-9. It is also worth noting that the fibronectin domain of MMP-9 does not participate in interactions with NGAL.

## Discussion

### Covalent and noncovalent MMP-9 HPX/NGAL complex

The obtained model of the covalent MMP-9 HPX complex with NGAL allowed for the identification of key interactions that stabilize its structure (Table 1 and Fig. 1). The complex exhibited 20 recurring residue–residue interaction pairs across successive simulations. From the perspective of complex formation, the most interesting interactions are those occurring near the disulfide bridge Cys674–Cys87. One of these is the hydrogen bond between His662 and Glu91. His662 is an amino acid whose side chain in the crystallographic structure shields Cys674 from solvent access. This type of interaction may be the direct cause of Cys674 exposure during protein recognition, just before the formation of the covalent bond. It is worth noting that the position of Glu91 is stabilized by a hydrogen bond and electrostatic interaction between Glu91–Arg109, as well as a hydrogen bond between Cys87 and Glu91, in which the main chain of Cys87 participates (Cys87, Glu91, and Arg109 belong to the NGAL protein). Another interaction located near the disulfide bridge linking the complex subunits is the hydrogen bond between Lys535 and Pro85. The Lys535 side chain is stabilized in this region by an electrostatic interaction with Asp522 and a hydrogen bond with the main chain of Ile520. Similar to Lys535, both of these amino acids belong to MMP-9.

**Fig. 3.**
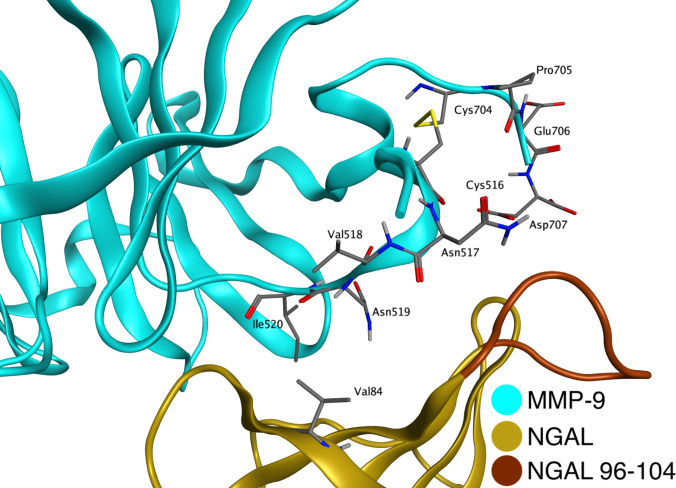
N-terminal region of the MMP-9 HPX in covalent complex with NGAL. The Asn96–Thr104 loop and Val84 of NGAL participate in the protein–protein interaction interface with the HPX of MMP-9 and are located in a position that may sterically hinder access to the hemopexin-domain cleavage site. The structure shown represents a representative snapshot from the equilibrated MD simulation.

The MMP-9/NGAL complex is associated with an increase of MMP-9 enzymatic activity. Commonly, 2 mechanisms are discussed—promotion of proMMP-9 autoactivation by NGAL and protection from degradation [15,16], although the exact cleavage site of the polypeptide chain is unknown. In the developed model, the N-terminus of the HPX is located in close proximity to NGAL, which may indicate the specific region performing the discussed protective functions (Fig. 3). This region mainly consists of the NGAL loop composed of amino acids Asn96–Thr104 and the single NGAL amino acid Val84. Although Val84 does not directly form contacts with MMP-9, it is positioned directly above the hydrophobic region of the HPX and may act as an effective steric hindrance to the binding of the degradative enzyme. In this way, the amino acids Asp513 to Phe521 are protected from proteolytic enzyme access.

Comparing the covalent and noncovalent MMP-9 HPX complexes with NGAL, no notable differences in their overall structure were observed. The interaction energy in the noncovalent MMP-9 HPX complex with NGAL is at a similar level to that in the system containing a disulfide bridge, indicating high stability of this complex on the time scale of the simulations. One should note, however, that this methodology does not include the energy of the S–S bond itself and that the purpose of comparing the covalent and noncovalent complexes is to evaluate the stability and organization of the protein–protein interface, not to quantitatively assess the energetic contribution of the disulfide bond. This is consistent with literature data showing that MMP-9 and NGAL can spontaneously form a complex, although this interaction is dynamic and transient [15]. At the same time, recent reports indicate that NGAL stabilizes MMP-9 and protects it from proteolytic degradation and that only a fraction of MMP-9 occurs in the form of a disulfide-linked MMP-9/NGAL complex [30].

The average contact surface between the subunits in systems with and without a covalent bond is also at a similar level. These results suggest that the disulfide bridge primarily serves to stabilize the complex geometry rather than directly increasing the interaction strength between the subunits. Importantly, this observation is consistent with evolutionary and functional data indicating that the disulfide bond in the human MMP-9/NGAL complex provides an additional level of stabilization rather than being a prerequisite for complex formation [31]. This implies that the removal of the disulfide bond does not lead to partial dissociation of the system, e.g., by disrupting contacts in a specific region. Such NGAL-dependent stabilization of MMP-9 may be beneficial for the regulation of host defense but may also contribute to detrimental pathological effects, particularly in inflammatory diseases [31].

Most interactions in the noncovalent complex correspond to those in the covalent form, although the absence of the disulfide bridge causes certain differences. The noncovalent complex preserves a highly stable hydrophobic interaction core at the MMP-9/NGAL interface, formed mainly by van der Waals contacts involving Ile520 of MMP-9 (Ile520–Gly86, Ile520–Thr93, and Ile520–Leu107), whose occupancies remain close to 100% in both systems. The hydrogen bond between Lys535 (MMP-9) and Pro85 (NGAL), located in the vicinity of the disulfide bridge, remains highly stable in both the covalent and noncovalent systems. A cluster of highly persistent electrostatic interactions involving residues Asp676 and Arg677 of the MMP-9 HPX and Lys124 and Glu131 of NGAL is fully conserved in the noncovalent complex, indicating that this region constitutes a stable anchoring site of the interface. In contrast, several hydrogen bonds show reduced occupancies in the noncovalent system, indicating a partial local reorganization of the interface upon removal of the disulfide bridge.

The observed stability and preservation of key interface features in the noncovalent MMP-9/NGAL complex indicate that this assembly may, similarly to the disulfide-linked complex, constitute a structurally reliable platform for further interactions with regulatory proteins, including the inhibitor TIMP-1. Despite the potentially transient nature of the MMP-9/NGAL interactions, the presence of additional regulatory partners such as TIMP-1 may further stabilize this assembly within more sophisticated protein complexes.

### Inhibitory MMP-9/NGAL/TIMP-1/MMP-9 tetramer

**Fig. 4.**
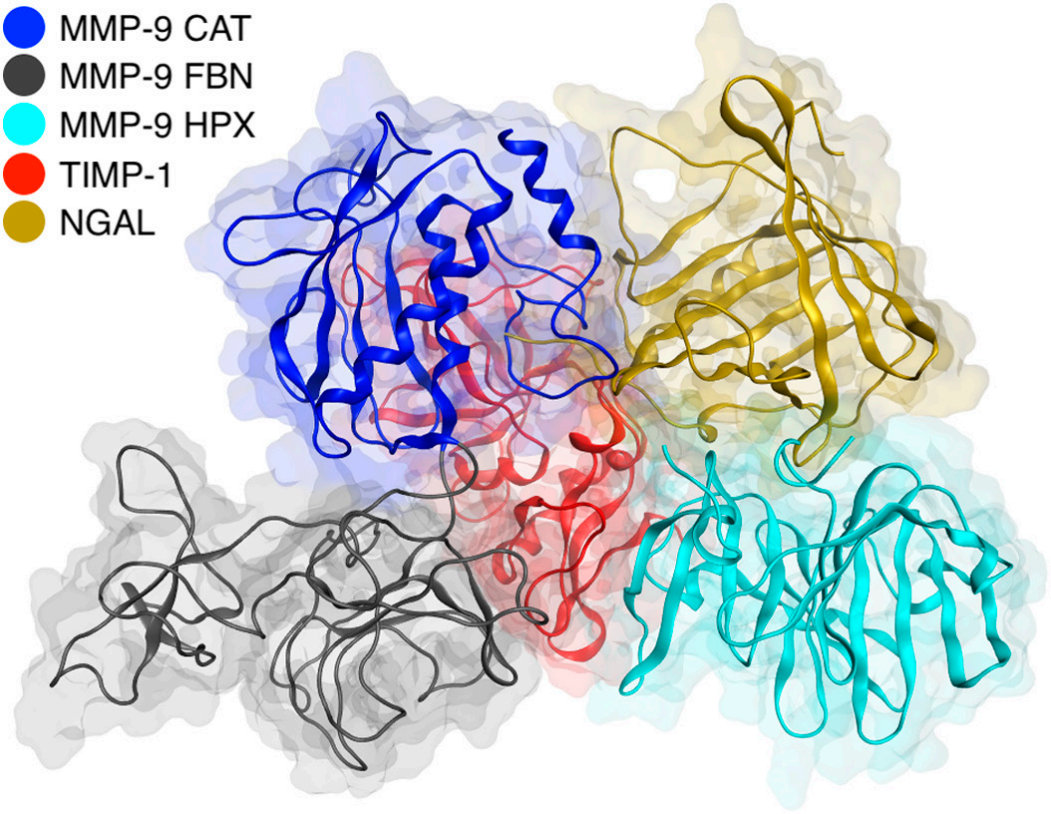
Relative orientation of MMP-9 domains and the NGAL and TIMP-1 proteins in the inhibitory tetrameric complex. In this view, the TIMP-1 molecule is located behind the other molecular components. The structure shown represents a representative snapshot from the equilibrated MD simulation.

As mentioned in the introduction, the proMMP-9/NGAL complex is naturally secreted by cells, mainly neutrophils and monocytes, in the form of a covalent complex [12,15]. Experimental studies indicate that TIMP-1 can also associate with the MMP-9/NGAL complex, which includes the HPX of MMP-9 [19], forming a more complex structure—a proMMP-9/NGAL/TIMP-1 trimer (Fig. 4). Moreover, it has been shown that the presence of NGAL increases the association constant of TIMP-1 to the HPX of MMP-9 [19]. This suggests an important role for NGAL in modulating the interaction between MMP-9 and its inhibitors, which may be relevant to the regulation of MMP-9 proteolytic activity under physiological and pathological conditions. In the present study, the regulatory role of NGAL is understood as its ability to modulate the stability, spatial organization, and accessibility for TIMP-1 binding, rather than as a direct inhibition of MMP-9 catalytic activity. An additional aspect of NGAL’s involvement is the ability of the proMMP-9/NGAL/TIMP-1 complex to inhibit another, already activated MMP-9 molecule. Such complexes have been observed and described in the literature as tetramers [19].

However, it should be noted that the presence of 2 MMP-9 molecules in the complex results directly from the experimental procedure, which involved combining a proMMP-9 fraction precomplexed with NGAL and TIMP-1 with a fraction of activated MMP-9. It is also worth mentioning that, in vitro, the proMMP-9/NGAL/TIMP-1 complex is capable of inhibiting MMP-3 as well [19]. Structural analysis does not indicate any obstacles to the formation of a similar complex involving only a single MMP-9 molecule. Moreover, from a biological perspective, such a configuration more closely resembles the typical MMP-9 processing pathway, in which TIMP-1 is prebound to the HPX of MMP-9 before its activation.

Laboratory studies of MMP-9 complexes with NGAL also demonstrate the possibility of forming complexes that include not only MMP-9, NGAL, and TIMP-1 but also MMP-2. Furthermore, such heterotetramers have been identified in cardiac tissue, both in normally functioning hearts and in those affected by cardiomyopathy [18]. The authors noted that the complexes are enzymatically inactive but showed that active forms of MMP-9 and MMP-2 can be spontaneously released from them. However, the exact mechanism of release and activation has not been described [18].

It should be emphasized that, despite the growing body of experimental data on MMP-9 and NGAL-containing complexes, the specific domains responsible for the interactions between individual components of these assemblies have not been identified. A detailed structural description of a HPX-mediated interaction between a metalloproteinase and its inhibitor is currently available only for the proMMP-2/TIMP-2 complex [32], whereas for MMP-9, the available experimental structures comprise only individual domains of this enzyme (according to the RCSB Protein Data Bank) [33]. Essentially, only a single detailed computational model of the MMP-9/TIMP-1 complex is available, in which TIMP-1 interacts with the catalytic, fibronectin-like, and HPXs of MMP-9, while earlier reports are limited to simplified docking analyses [24]. Importantly, structural and sequence analyses of the proMMP-2/TIMP-2 complex [32] provide molecular insight into the specificity of MMP-2 interactions with TIMP-2, TIMP-3, and TIMP-4 and, indirectly, of the MMP-9/TIMP-1 pair.

The crystal structure of the proMMP-2/TIMP-2 complex demonstrated that the interaction is mediated solely by the HPX of MMP-2 and the C-terminal domain of TIMP-2, while the catalytic site of the enzyme and the inhibitory region of the inhibitor remain spatially separated. On this basis, TIMP-2 was proposed to function as a docking and bridging component enabling the formation of higher-order regulatory complexes rather than acting only as a classical inhibitor of proteolytic activity. This regulatory mode provides a direct structural analogy for the role proposed here for NGAL in MMP-9-containing assemblies. In turn, the involvement of the HPX of MMP-9 in the formation of ternary regulatory assemblies comprising TIMP-1 and MMP-3 has been demonstrated [34].

Taken together, these observations indicate that the HPX may serve as a versatile protein–protein interaction platform that promotes the formation of higher-order regulatory complexes. Coevolutionary analyses indicate that the MMP-9/NGAL interaction has emerged during primate evolution and involves primarily residues located within the HPX of MMP-9 [31], further supporting the functional relevance of the HPX-mediated interface observed in our structural models. The proposed architecture of the MMP-9/NGAL and MMP-9/NGAL/TIMP-1 complexes may therefore represent a regulatory mechanism that evolved specifically in primates. Notably, the main interaction hotspot identified in our simulations within the MMP-9 HPX (residues 660 to 677), comprising, among others, Asp660, His662, and Cys674–Arg677, overlaps with the region (residues 665 to 675) previously identified as coevolving with NGAL in sequence-based analyses [31].

In this context, the MMP-9/NGAL/TIMP-1/MMP-9 architecture proposed in the present study fits well within the concept of previously described MMP-9-centered regulatory assemblies. Moreover, it has been shown that the binding interface of TIMP-1 is intrinsically flexible, enabling its conformational adaptation to different metalloproteinase partners and distinct complex architectures [35,36].

It should be noted, however, that a direct interaction between the HPX of MMP-9 and TIMP-1 in the absence of NGAL is a predominant complex form, which is similar to proMMP-2/TIMP-2 structure. In the noninhibitory proMMP-2/TIMP-2 regulatory complex, the inhibitor surface remains largely accessible for canonical binding to the catalytic domain of another metalloproteinase. Accordingly, our models indicate that NGAL primarily acts as a structural regulator that modulates the stability and spatial organization of the MMP-9/TIMP-1 complex, rather than being a prerequisite for the interaction between these 2 proteins. It should be noted that the presented models describe the architecture and stability of protein assemblies and do not directly address the role of metal cofactors required for MMP-9 catalytic activity; therefore, any potential functional implications should be interpreted with caution.

Importantly, clinical and translational studies indicate that MMP-9, NGAL, and TIMP-1 are each independently associated with inflammatory processes and tissue injury in several pathological conditions, including inflammatory bowel disease and microvascular complications of diabetes [37,38]. Although direct clinical evidence for a simultaneous increase of all 3 components within a single patient cohort is currently lacking, available data demonstrate functional interactions within this system, including the formation of the MMP-9/NGAL complex and the tight regulation of MMP-9 activity by TIMP-1. Together, these findings support the concept of a regulatory MMP-9–NGAL–TIMP-1 network involved in inflammatory responses and tissue injury.

In contrast to studies assessing individual components of this system, direct evidence for the presence of circulating MMP-9/NGAL complexes is provided by clinical studies based on analyses of patient serum [39]. Notably, the circulating MMP-9/NGAL complex displays a strong dependence on patient-related biological context, including age, metabolic status, and tumor molecular subtype, indicating that the formation and systemic availability of this assembly are tightly regulated and disease-context dependent.

In the model of the MMP-9/NGAL/TIMP-1/MMP-9 complex presented here, one of the MMP-9 molecules—the one containing the HPX—can exist in either a latent or active state. This, together with the previously described shielding of the C-terminal region of the HPX from degradation [15], suggests that the secreted proMMP-9/TIMP-1 complex may serve as a reservoir of MMP-9 in the extracellular space. The presence of NGAL enhances its resistance to degradation, enabling longer retention in tissue, while maintaining the potential for proteolytic activation and inhibition via TIMP-1 prebound to the HPX.

Clinical observations indicate that an early postoperative and inflammation-associated increase in MMP-9 and TIMP-1 levels correlates with the extent of subsequent tissue damage and functional deterioration [40]. These observations support the concept that the organization and stabilization of MMP-9-containing complexes, rather than the mere presence of the enzyme or its inhibitor, may be important for regulating the availability and spatial distribution of MMP-9 in tissues in vivo.

Additional interactions between NGAL and TIMP-1 clearly explain the observed increase in TIMP-1 affinity for the MMP-9/NGAL complex compared to the NGAL-free system. This interaction appears to be important in protecting the complex from degradation, consistent with the protective effect conferred by NGAL binding. If such a form of MMP-9 were secreted from the cell without a bound TIMP-1 and subsequently activated, it could lead to uncontrolled proteolysis. The presence of NGAL, by stabilizing the structure and increasing TIMP-1 affinity for MMP-9 at the latent stage, may act as a safeguard mechanism. The increased association constant with the inhibitor promotes early binding, thereby enhancing the likelihood of effective inhibition following enzyme activation and release into the extracellular space.

In this context, literature data indicate that the intrinsic flexibility of TIMP-1 and stabilization of its internal hydrogen-bond network affect the association process with metalloproteinases, primarily by reducing the entropic cost of complex formation and increasing the association rate [35]. This mechanism, based on precise control of active MMP-9 availability, may be of particular importance in pathologies associated with excessive proteolysis, such as chronic inflammation, cancer, or tissue damage. Clinical data demonstrating the presence of circulating MMP-9/NGAL complexes in patients with inflammatory and neoplastic diseases suggest that the formation of such assemblies may be linked to altered systemic availability of MMP-9 under pathological conditions [39,41]. Importantly, circulating MMP-9/TIMP-1 complexes have also been detected in the plasma of cancer patients, indicating that different functional forms of MMP-9 complexes may coexist in the systemic circulation under pathological conditions [42]. A better understanding of the structural basis of NGAL-dependent regulation of MMP-9 assemblies may, in the future, support the design of more selective therapeutic strategies aimed at modulating MMP-9 activity without the need for direct catalytic inhibition.

## Conclusions

Our results demonstrate that both covalent and noncovalent MMP-9/NGAL complexes display comparable interaction energies and contact surfaces, indicating that the spontaneously formed noncovalent complex is structurally stable. Interaction mapping allowed us to define an NGAL-protected region of the HPX (Asp513–Phe521), providing a structural explanation for the experimentally observed resistance to autodegradation. The tetrameric MMP-9/NGAL/TIMP-1/MMP-9 model further shows that NGAL does not disrupt TIMP-1 binding and contributes to the structural organization of the inhibitor within the complex through additional interaction interfaces. These findings provide structural insight into NGAL-mediated modulation of MMP-9 assemblies and support therapeutic concepts aimed at stabilizing latent MMP-9-containing complexes rather than directly blocking the catalytic site. Overall, the present results indicate that NGAL, while not required for MMP-9–TIMP-1 association, modulates the stability and architecture of MMP-9-centered regulatory complexes.
