# Supplementary material for: Exploring the Regulatory Function of NGAL in MMP-9 Complexes
Source: Comput Struct Biotechnol J. 2026 Mar 30;35(1):0006. doi: 10.34133/csbj.0006 (PMC13082463; doi:10.34133/csbj.0006)
Supplement: Supplementary 1 — Figs. S1 to S9 [file csbj.0006.f1.docx]

S.1. Computational workflow

The overall computational workflow consisted of the following steps:

1. **Structure acquisition**The crystallographic structures of NGAL (PDB ID: 1DFV, chain A) and the MMP-9 hemopexin domain (PDB ID: 1ITV, chain A) were obtained from the Protein Data Bank in PDB format.
2. **Initial structure preparation**The NGAL structure was prepared by removing the covalently linked saccharide moiety. The MMP-9 hemopexin structure was prepared by minor manual adjustments of the Gln675–Arg677 region and rotation of the His662 side chain in order to expose the Cys674 residue.
3. **Energy optimization of input structures**Both protein structures were energy-minimized using the AMBER10:EHT force field in MOE2020 in order to remove local steric clashes and obtain relaxed starting geometries for docking.
4. **Protein-protein docking**

Protein-protein docking of NGAL and the MMP-9 hemopexin domain was performed in MOE2020. The aim of this step was to identify plausible binding modes and candidate protein-protein interfaces.

1. **Construction of covalent and non-covalent MMP-9/NGAL complexes**The best-scored docking model was used to construct a disulfide-linked MMP-9/NGAL complex by applying distance restraints between Cys674 (MMP-9) and Cys87 (NGAL), followed by energy minimization and explicit introduction of the S–S bond. The corresponding non-covalent complex was obtained by removal of the disulfide bond and subsequent minimization**.**
2. **Modeling of the higher-order assembly**The MMP-9/NGAL/TIMP-1/MMP-9 tetramer was constructed using a previously published model of the terminal inhibitory MMP-9/TIMP-1 complex, followed by structural alignment of the covalent MMP-9/NGAL complex and removal of steric clashes.
3. **Molecular dynamics simulations**All modeled systems were solvated explicitly and subjected to energy minimization and 10 ns molecular dynamics simulations in NAMD in order to relax the structures and assess the short-timescale stability of the interfaces.
4. **Trajectory analysis and interaction characterization**The resulting trajectories were analyzed to determine RMSD, RMSF, interfacial contact frequencies and interaction energies. All interaction and comparison analyses were performed on equilibrated trajectory frames after RMSD stabilization, and not on the initial docking poses.

## S.2. Covalent MMP-9 HPX/NGAL complex


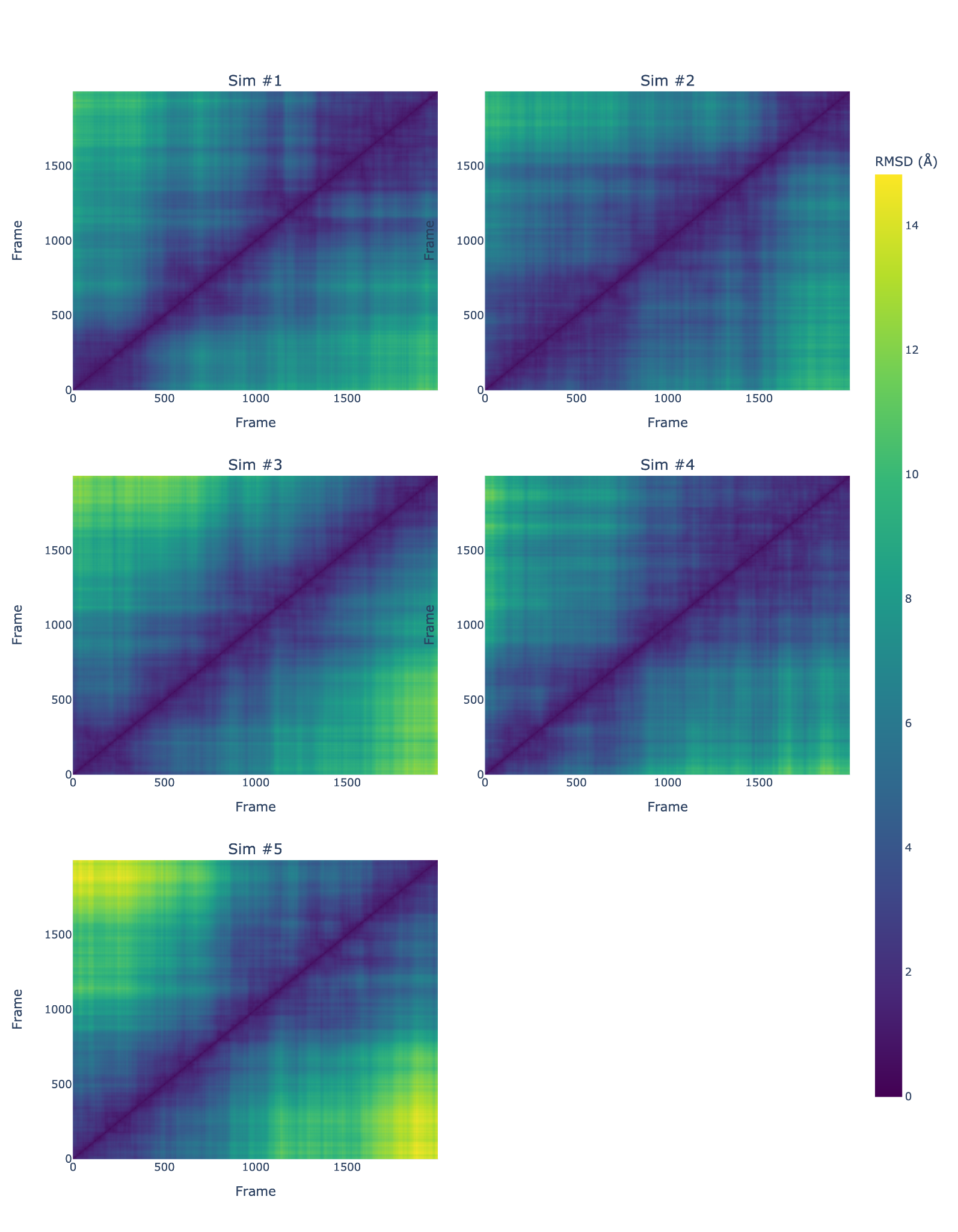


Figure S1. Pairwise frame-to-frame RMSD matrices calculated on Cα atoms for five independent MD replicas of the covalent MMP-9 HPX/NGAL dimer complex (Sim #1–Sim #5). Trajectory frames were saved every 5 ps. The color scale represents RMSD values.


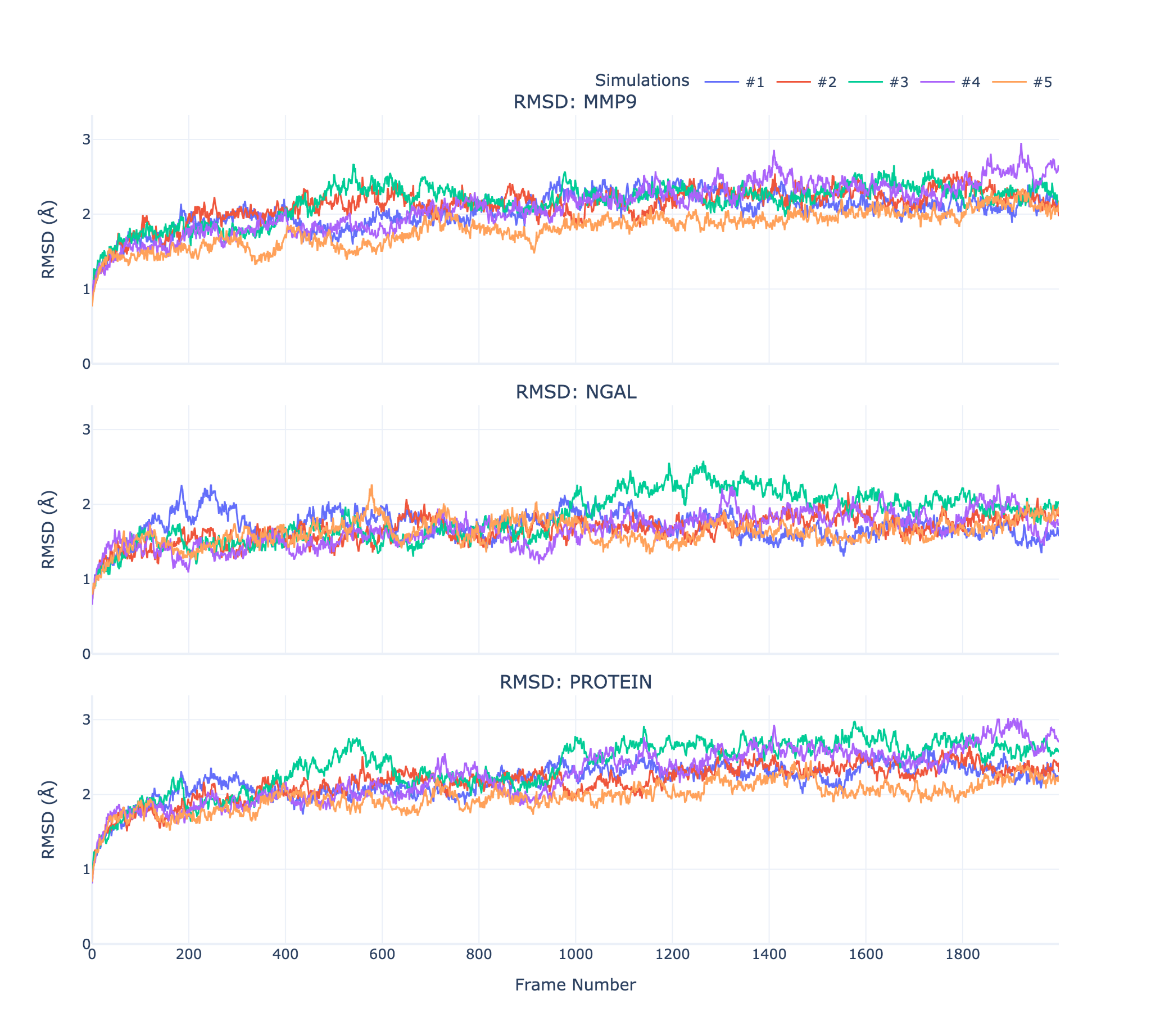


Figure S2. RMSD profiles as a function of simulation frame calculated for the protein backbone atoms (excluding hydrogens) for five independent MD replicas (Sim#1 – Sim#5) of the covalent MMP-9 HPX/NGAL dimer complex. RMSD is shown separately for the HPX domain of MMP-9 (denoted as MMP9), for NGAL, and for the entire MMP-9 HPX/NGAL dimer (PROTEIN). Trajectory frames were saved every 5 ps. Individual curves correspond to independent simulation replicas and are color-coded according to the legend shown above the plots.

The pairwise frame-to-frame RMSD matrices calculated for Cα atoms show a consistent overall conformational pattern among the independent simulations of the covalent MMP-9 HPX/NGAL dimer, with increased RMSD values mainly between temporally distant frames. The RMSD time profiles for the hemopexin domain of MMP-9, for NGAL, and for the complete dimer indicate stable internal structures of both subunits and moderate fluctuations of their relative arrangement. Importantly, no systematic increase of RMSD with simulation time is observed for any replica, indicating the absence of progressive structural drift of the complex during the analyzed trajectories.


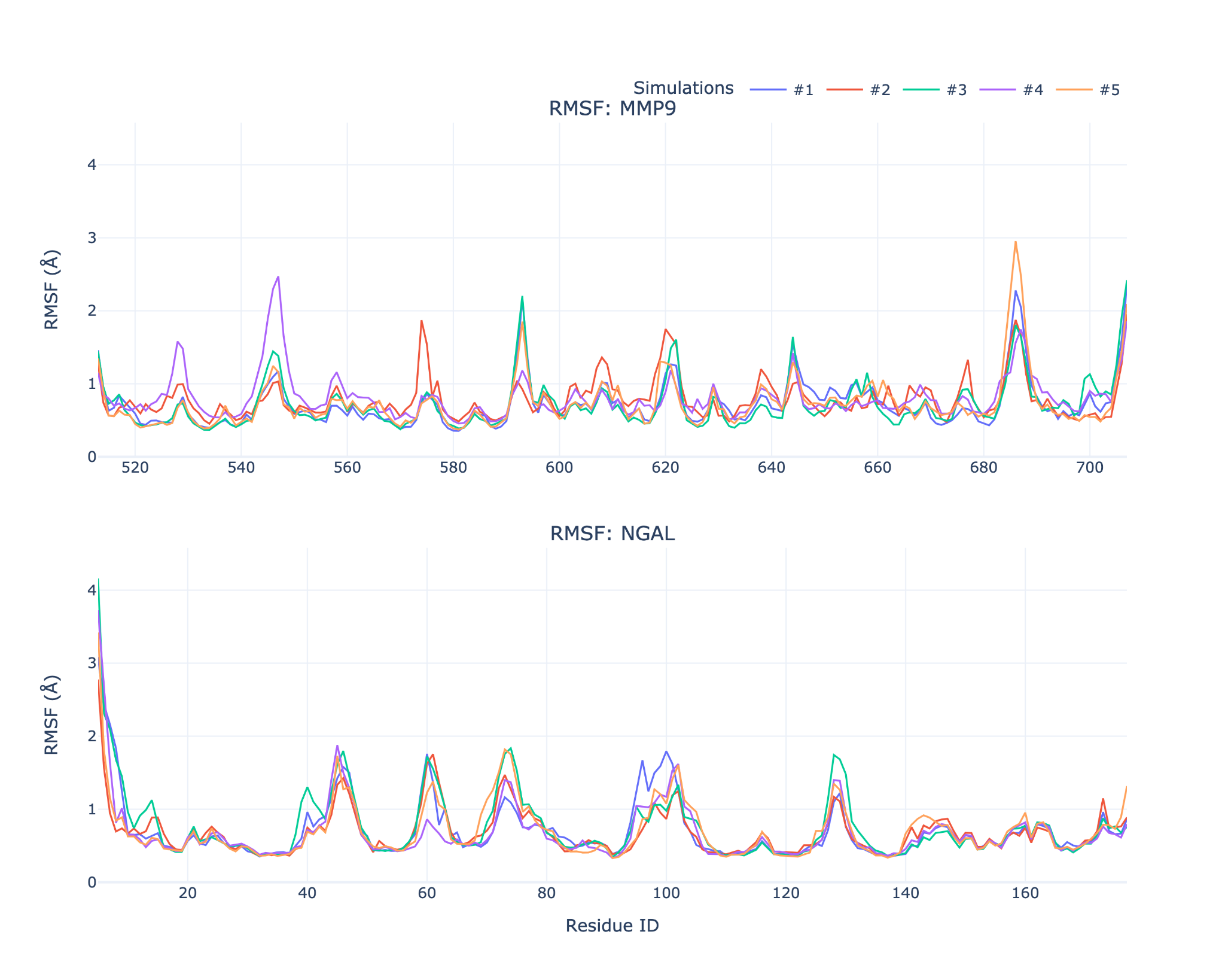


Figure S3. RMSF profiles calculated on Cα atoms for NGAL and the HPX domain of MMP-9 from five independent MD replicas (Sim #1–Sim #5) of the covalent MMP-9 HPX/NGAL dimer complex. Individual curves correspond to independent simulation replicas and are color-coded according to the legend.

The RMSF profiles for the MMP-9 hemopexin domain and NGAL are largely consistent with the contact occupancy analysis. Residue pairs classified as stable (≥70%) or frequent (≥50%) according to the adopted criteria (e.g. Ile520-Gly86/Thr93/Leu107, Asp676-Lys124, Arg677-Glu131 and Lys535-Pro85) are located predominantly in regions of low to moderate RMSF, indicating local stabilization of the HPX/NGAL interface.

In contrast, residue pairs classified as intermittent (≥20%) or transient (≥10%) (e.g. interactions involving Asn517, Asn519, Gln675 and His662) correspond to regions displaying elevated RMSF values, suggesting that these contacts are formed mainly by flexible interface loops and have a dynamic, short-lived character.

The highest RMSF values, observed mainly at the chain termini, particularly at the N-terminus of NGAL, do not coincide with residue pairs classified as stable or frequent and therefore reflect intrinsic flexibility of regions that do not constitute the core of the MMP-9 HPX/NGAL interface. Overall, the combined RMSF and contact occupancy analyses consistently indicate a stable central interface between MMP-9 hemopexin domain and NGAL, surrounded by more flexible peripheral regions forming intermittent and transient contacts.

## S.3. Non-covalent MMP-9 HPX/NGAL complex


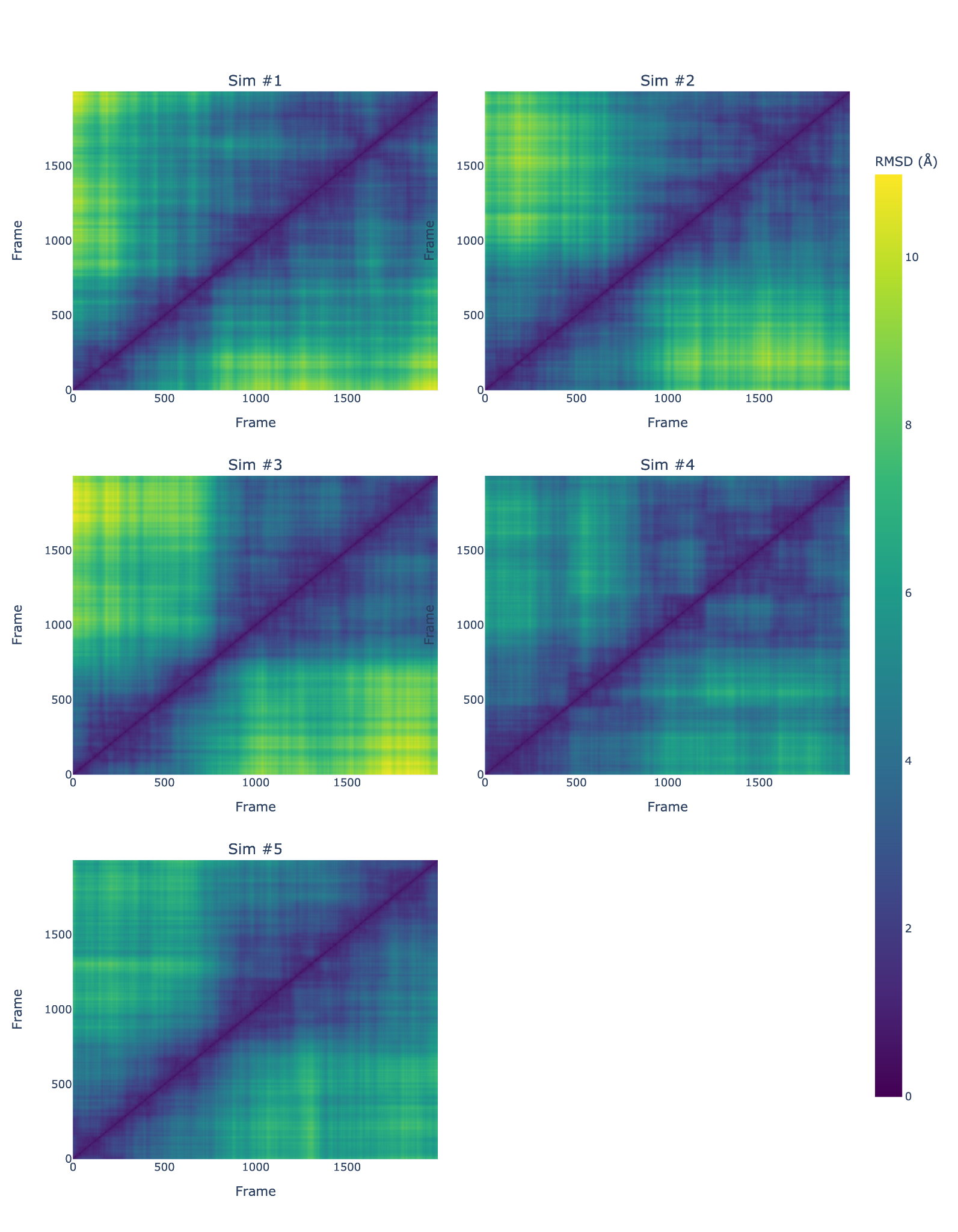


Figure S4. Pairwise frame-to-frame RMSD matrices calculated on Cα atoms for five independent MD replicas of the non-covalent MMP-9 HPX/NGAL dimer complex (Sim#1 - Sim#5). Trajectory frames were saved every 5 ps. The color scale represents RMSD values.


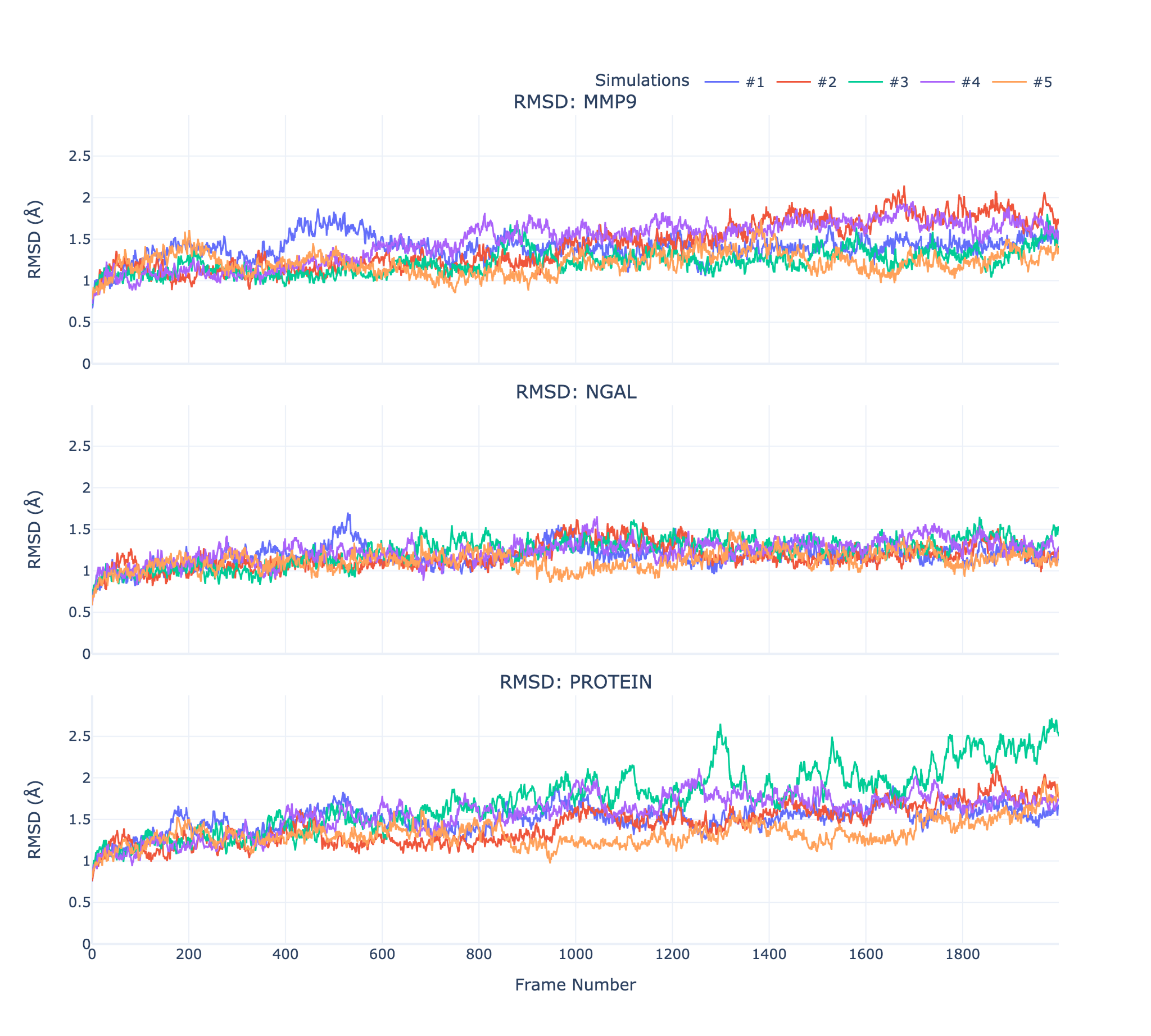


Figure S5. RMSD profiles as a function of simulation frame calculated for the protein backbone atoms (excluding hydrogens) for five independent MD replicas (Sim#1 - Sim#5) of the non-covalent MMP-9 HPX/NGAL dimer complex. RMSD is shown separately for the HPX domain of MMP-9 (denoted as MMP9), for NGAL, and for the entire MMP-9 HPX/NGAL dimer (PROTEIN). Trajectory frames were saved every 5 ps. Individual curves correspond to independent simulation replicas and are color-coded according to the legend shown above the plots.

The pairwise frame-to-frame RMSD matrices calculated on Cα atoms for the non-covalent MMP-9 HPX/NGAL complex show a broadly consistent conformational pattern across the independent simulations, with higher RMSD values mainly observed for pairs of frames separated by longer time intervals.

The RMSD time profiles of the individual subunits (the MMP-9 hemopexin domain and NGAL) indicate stable internal structures after the initial relaxation, whereas the RMSD of the whole complex reflects moderate fluctuations of the relative arrangement of the two proteins.

Importantly, no systematic increase of RMSD with simulation time is observed for any replica, indicating the absence of progressive structural drift of the non-covalent complex and supporting its overall structural stability within the analyzed time window.

The overall qualitative behavior of the covalent complex is comparable to that observed for the non-covalent system.


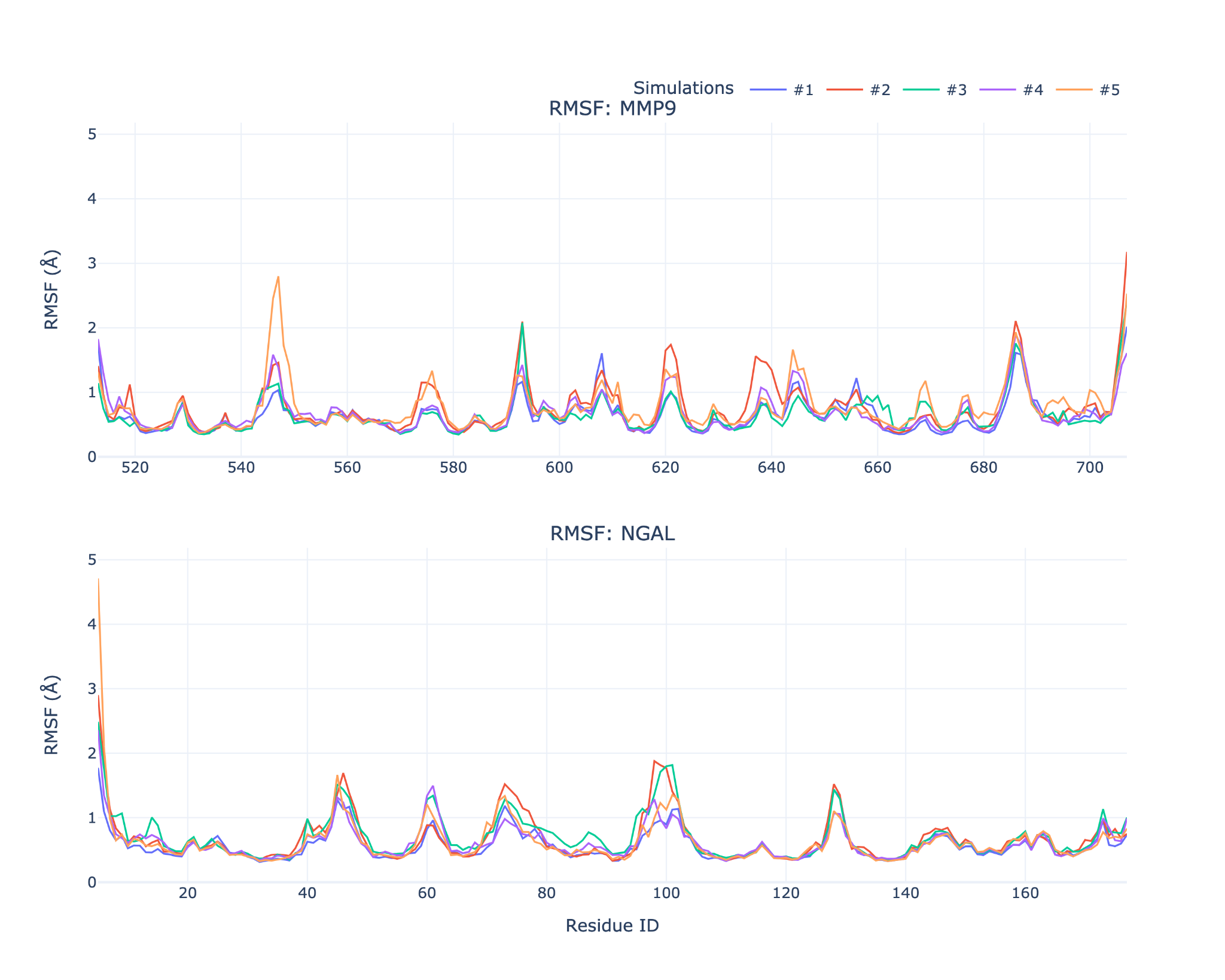


Figure S6. RMSF profiles calculated on Cα atoms for NGAL and the HPX domain of MMP-9 from five independent MD replicas (Sim#1 - Sim#5) of the non-covalent MMP-9 HPX/NGAL dimer complex. Individual curves correspond to independent simulation replicas and are color-coded according to the legend.

The RMSF profiles calculated after 5 ns show that both the MMP-9 hemopexin domain and NGAL remain globally stable, with increased flexibility restricted mainly to loop regions and to the N-terminus of NGAL. The residues forming the core of the MMP-9 HPX/NGAL interface display low to moderate RMSF values, indicating locally stabilized regions at the binding interface.

Consistently, the contact occupancy analysis identifies a set of highly persistent interactions in the non-covalent system (in particular contacts involving Ile520, Asp676–Lys124 and Arg677–Glu131), which coincide with regions of reduced atomic fluctuations. In contrast, contacts classified as intermittent or transient are predominantly associated with residues located in flexible loop segments, as reflected by locally elevated RMSF values.

**Conclusions**

The RMSD profiles of the hemopexin domain of MMP-9, NGAL and the full MMP-9 HPX/NGAL complex show comparable ranges of structural fluctuations in the covalent and non-covalent systems. In both cases, the RMSD time series display an initial relaxation followed by stable fluctuations, and no systematic increase of RMSD with simulation time is observed in any replica.

The RMSF profiles calculated for Cα atoms reveal very similar flexibility patterns in the two systems. In both complexes, enhanced atomic fluctuations are mainly localized in loop regions and at the N-terminus of NGAL, whereas residues forming the MMP-9 HPX/NGAL interface exhibit predominantly low to moderate RMSF values.

Overall, the RMSD and RMSF analyses indicate that removal of the disulfide bond does not induce major changes in the global structural behavior or in the distribution of local flexibility of the MMP-9 HPX/NGAL complex within the analyzed time window.

## S.4. Inhibitory MMP-9/NGAL/TIMP-1/MMP-9 tetramer


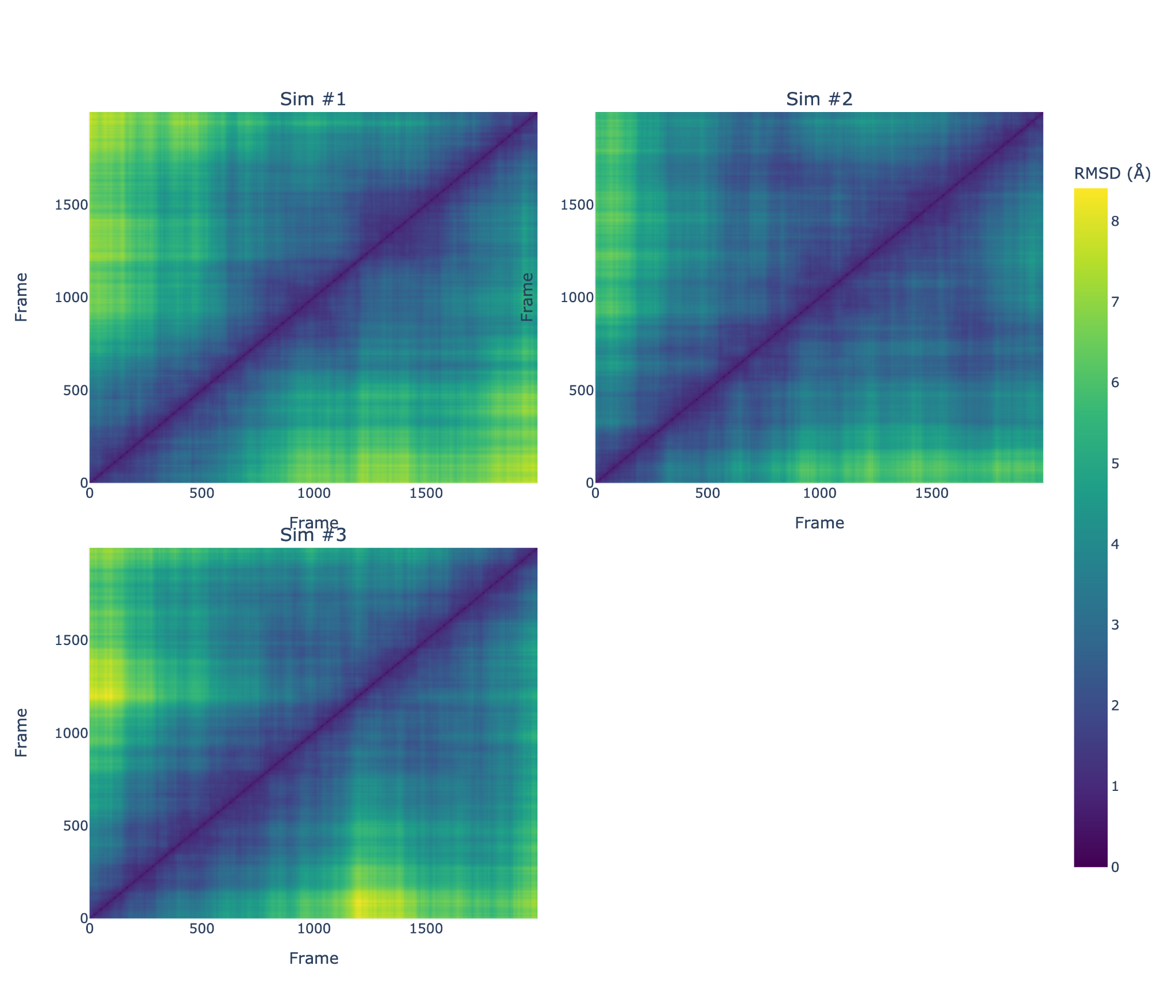


Figure S7. Pairwise frame-to-frame RMSD matrices calculated on Cα atoms for independent MD replicas of the inhibitory MMP-9/NGAL/TIMP-1/MMP-9 tetramer complex (Sim#1 - Sim#3). Trajectory frames were saved every 5 ps. The color scale represents RMSD values.

The pairwise RMSD matrices indicate substantial structural variability between distant trajectory frames in all three independent simulations of the multi-protein system. RMSD profiles show preserved internal structures of the individual components (HPX, NGAL and TIMP-1), whereas the CATFBN subcomplex, analyzed here as a single molecular fragment (because the fibronectin domain constitutes an insertion within the catalytic domain), exhibits increased variability compared with the components listed above. This is consistent with the high intrinsic flexibility of the fibronectin domain, and therefore constructs containing this fragment also display elevated RMSD values. The RMSD profile labeled as PROTEIN refers to the entire analyzed system comprising all components included in the simulation (CAT, FBN, HPX, NGAL and TIMP-1). The observed behavior reflects both the intrinsic flexibility of the fibronectin domain and the relative motions of the CATFBN fragment with respect to the remaining components of the complex, without evidence of progressive structural drift.


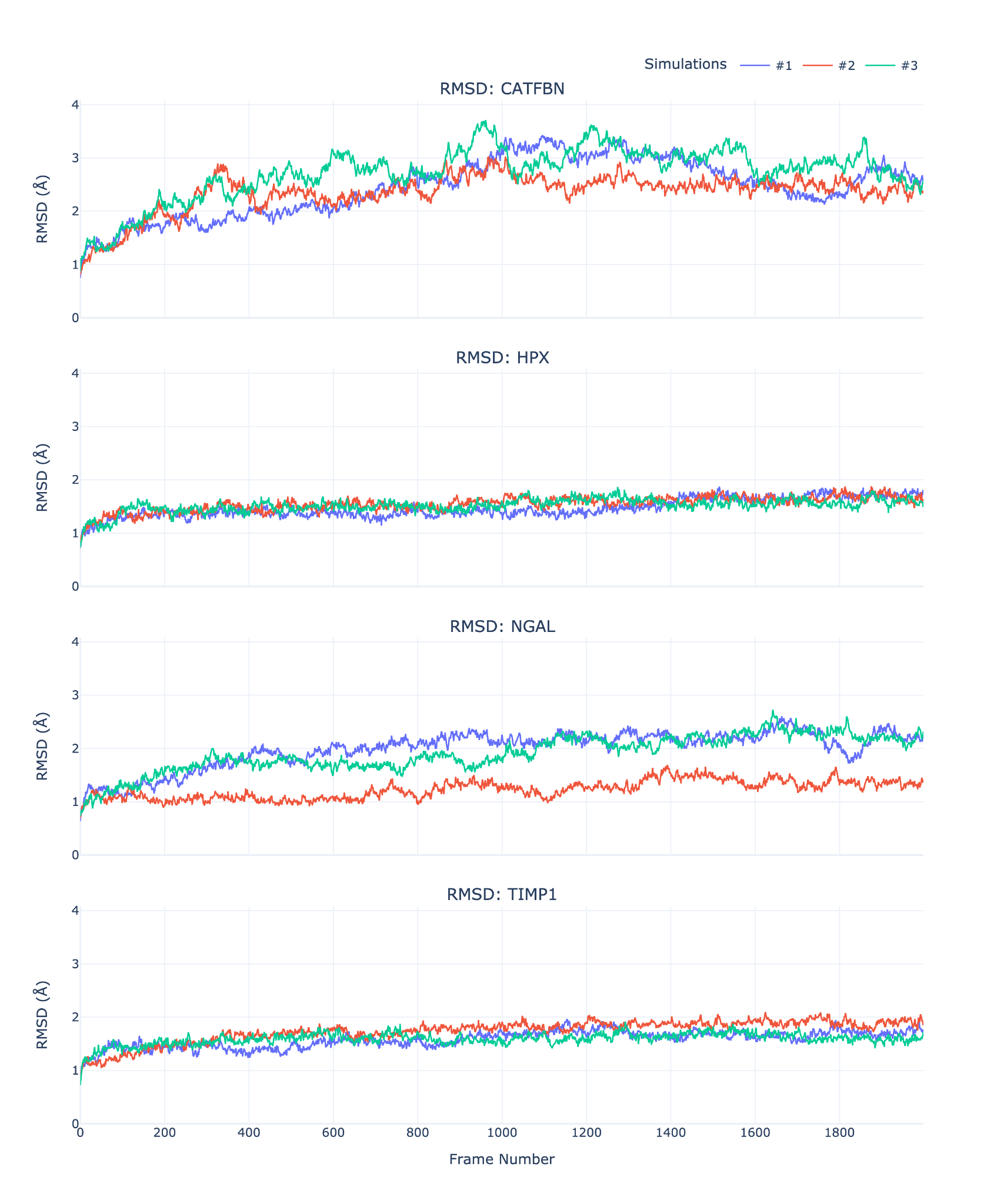


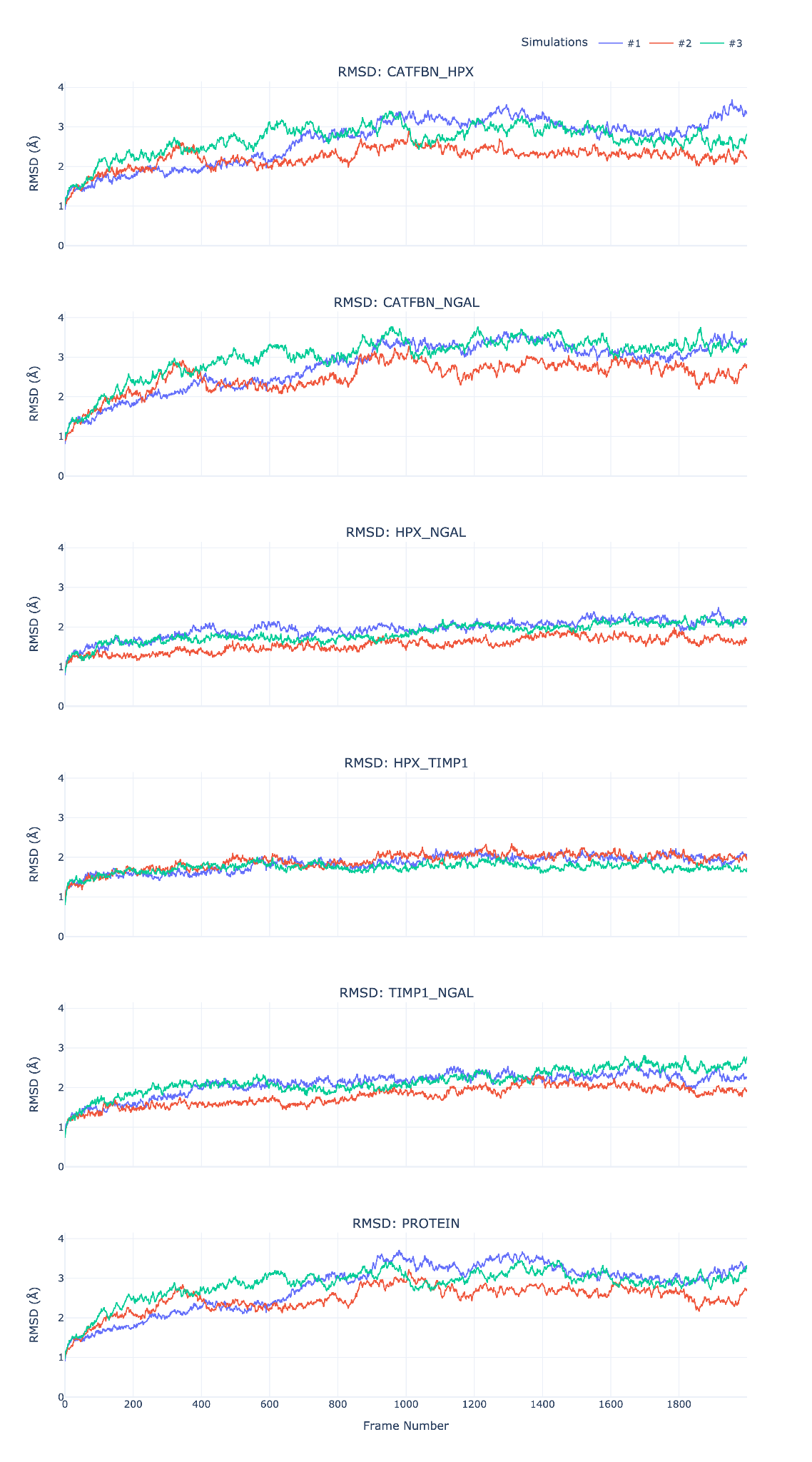


Figure S8. RMSD profiles as a function of simulation frame calculated for protein backbone atoms (excluding hydrogens) for three independent MD replicas (Sim#1 - Sim #3) of the inhibitory MMP-9/NGAL/TIMP-1/MMP-9 multi-protein complex. RMSD is shown separately for the HPX domain of MMP-9, NGAL and TIMP-1, as well as for the CATFBN fragment treated as a single molecular unit (the fibronectin domain constitutes an insertion within the catalytic domain). The RMSD profile labeled as PROTEIN refers to the entire analyzed system comprising all components included in the simulation (CAT, FBN, HPX, NGAL and TIMP-1). Trajectory frames were saved every 5 ps. Individual curves correspond to independent simulation replicas and are color-coded according to the legend shown above the plots.


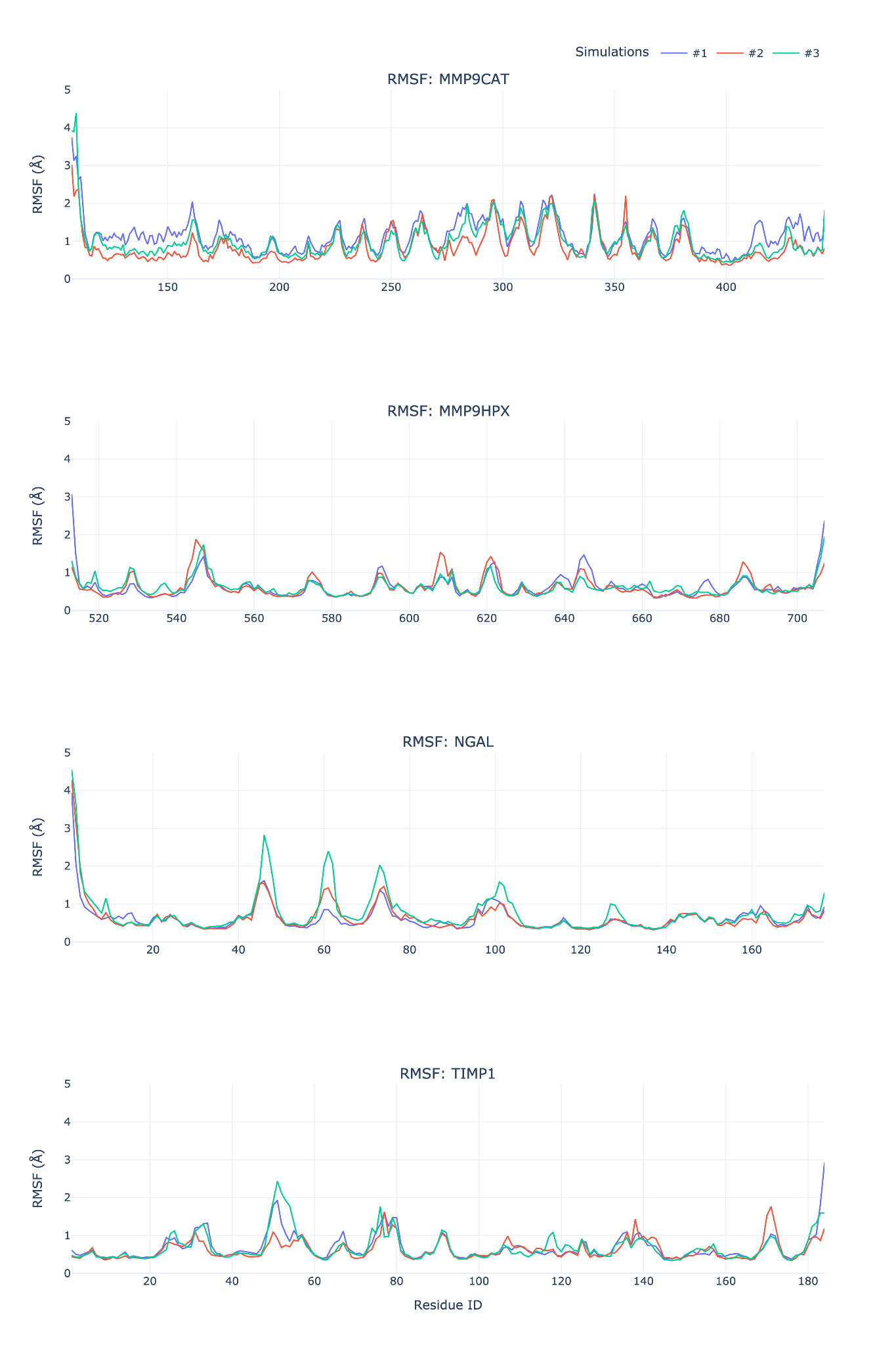


Figure S9. RMSF profiles calculated on Cα atoms for three independent MD replicas (Sim#1 - Sim#3) of the non-covalent MMP-9/NGAL/TIMP-1 multi-protein complex. RMSF is shown separately for the catalytic domain of MMP-9 (MMP9CAT), the hemopexin domain of MMP-9 (MMP9HPX), NGAL and TIMP-1. Individual curves correspond to independent simulation replicas and are color-coded according to the legend.

The RMSF profiles of the tetramer indicate that the highest local flexibility is associated with the MMP9CAT fragment (the catalytic domain together with the fibronectin insert), whereas the HPX domain, NGAL, and TIMP-1 remain clearly more rigid, with increased mobility mainly confined to loop regions and terminal segments. Comparison of the RMSF profiles with the contact analysis shows that the most persistent interactions, particularly hydrophobic contacts and key electrostatic pairs, are located in structurally rigid regions, supporting their role as a stable core of the protein-protein interfaces.

The MMP-9/NGAL interface is dominated by a highly stable hydrophobic core (notably involving Pro415 and Ile520) and a conserved cluster of electrostatic interactions within the HPX domain, whereas the NGAL–TIMP-1 contacts are predominantly frequent or transient and are concentrated in the N-terminal region of NGAL. Overall, the observed structural variability of the tetramer arises mainly from the intrinsic flexibility of the MMP9CAT fragment, while the principal protein-protein interfaces remain structurally stable.
